# Human blood MAIT cell subsets defined using MR1 tetramers

**DOI:** 10.1111/imcb.12021

**Published:** 2018-03-25

**Authors:** Nicholas A Gherardin, Michael NT Souter, Hui‐Fern Koay, Kirstie M Mangas, Torsten Seemann, Timothy P Stinear, Sidonia BG Eckle, Stuart P Berzins, Yves d'Udekem, Igor E Konstantinov, David P Fairlie, David S Ritchie, Paul J Neeson, Daniel G Pellicci, Adam P Uldrich, James McCluskey, Dale I Godfrey

**Affiliations:** ^1^ Department of Microbiology and Immunology Peter Doherty Institute for Infection and Immunity University of Melbourne Melbourne VIC 3000 Australia; ^2^ ARC Centre of Excellence in Advanced Molecular Imaging University of Melbourne Parkville VIC 3010 Australia; ^3^ Life Sciences Computation Centre Victorian Life Sciences Computation Initiative Carlton VIC 3053 Australia; ^4^ Federation University Australia Ballarat VIC 3350 Australia; ^5^ Fiona Elsey Cancer Research Institute Ballarat VIC 3350 Australia; ^6^ Royal Children's Hospital Flemington Road Parkville VIC 3052 Australia; ^7^ Division of Chemistry & Structural Biology Institute for Molecular Bioscience The University of Queensland Brisbane QLD 4072 Australia; ^8^ ARC Centre of Excellence in Advanced Molecular Imaging University of Queensland Brisbane QLD 4072 Australia; ^9^ Cancer Immunology Program Peter MacCallum Cancer Centre East Melbourne VIC 3002 Australia; ^10^ Department of Medicine University of Melbourne Parkville VIC 3010 Australia

**Keywords:** Human immunology, MAIT, MR1, T cell, unconventional T cell

## Abstract

Mucosal‐associated invariant T (MAIT) cells represent up to 10% of circulating human T cells. They are usually defined using combinations of non‐lineage‐specific (surrogate) markers such as anti‐TRAV1‐2, CD161, IL‐18Rα and CD26. The development of MR1‐Ag tetramers now permits the specific identification of MAIT cells based on T‐cell receptor specificity. Here, we compare these approaches for identifying MAIT cells and show that surrogate markers are not always accurate in identifying these cells, particularly the CD4^+^ fraction. Moreover, while all MAIT cell subsets produced comparable levels of IFNγ, TNF and IL‐17A, the CD4^+^ population produced more IL‐2 than the other subsets. In a human ontogeny study, we show that the frequencies of most MR1 tetramer^+^ MAIT cells, with the exception of CD4^+^ MAIT cells, increased from birth to about 25 years of age and declined thereafter. We also demonstrate a positive association between the frequency of MAIT cells and other unconventional T cells including Natural Killer T (NKT) cells and Vδ2^+^ γδ T cells. Accordingly, this study demonstrates that MAIT cells are phenotypically and functionally diverse, that surrogate markers may not reliably identify all of these cells, and that their numbers are regulated in an age‐dependent manner and correlate with NKT and Vδ2^+^ γδ T cells.

## Introduction

Mucosal‐associated invariant T (MAIT) cells are an evolutionarily conserved subset of unconventional T cells,^1^ restricted to the monomorphic major‐histocompatibility complex (MHC) class I‐like antigen (Ag)‐presenting molecule, MHC‐related protein 1 (MR1).^2^ MAIT cells are highly abundant in humans where they make up 1–10% of the circulating T cell pool,^3^, ^4^ and they are enriched in the liver^5^, ^6^ and at mucosal surfaces such as the gut.^2^, ^6^, ^7^

Human MAIT cells are also defined by expression of a semi‐invariant T‐cell receptor (TCR) composed of a very limited TCR‐α repertoire, comprising TRAV1‐2 joined to either TRAJ33,^8^, ^9^ TRAJ12 or TRAJ20,^7^, ^10^ with few, or no, n‐nucleotide additions at the Complementarity Determining Region (CDR)‐3α.^9^ These TCR‐α chains pair with a constrained repertoire of TCR‐β chains, predominantly utilizing the TRBV6 and TRBV20 gene families.^7^, ^9^, ^10^ In addition to their restriction to MR1, the MAIT TCR imbues MAIT cells with the unique ability to recognize a series of Ags derived from microbial riboflavin (vitamin B2) synthesis,^11^ the most potent of which is 5‐(2‐oxopropylideneamino)‐6‐D‐ribitylaminouracil (5‐OP‐RU).^12^ These Ags are produced by a wide array of bacteria and yeast species, all of which encode the riboflavin metabolic pathway.^4^, ^11^, ^13^ Thus, these riboflavin‐derivatives represent a molecular signature of microbial infection that can activate MAIT cells. Recently, however, a minor subset of MAIT cells was shown to detect folate (vitamin‐B9)‐based Ags presented by MR1,^14^ suggesting that MAIT cell subsets may also elicit TCR‐mediated responses in the absence of riboflavin‐derivatives.

MAIT cell development occurs in the thymus^2^ where immature thymocytes expressing MAIT TCRs interact with MR1‐expressing CD4^+^CD8^+^ double positive (DP) thymocytes.^15^ This evokes a three‐stage intrathymic developmental pathway after which mature MAIT cells egress to the peripheral circulation and tissues.^16^ There, they then expand in response to peripheral flora, and are maintained at high proportions in the circulation.^3^, ^16^ This combination of developmental cues is distinct from those followed by conventional T cells and results in the acquisition of a unique transcriptional profile. This includes an effector memory phenotype,^3^, ^6^ expression of the innate transcription factor promyelocytic leukemia zinc finger (PLZF), as well as RAR‐related orphan receptor gamma (RORγt) and intermediate levels of the T‐Box transcription factor TBX21 (T‐bet) in humans^17^, ^18^ or mutually exclusive RORγt^+^ or T‐bet^+^ subsets in mice.^19^ MAIT cells express tissue homing receptors^6^ and high levels of surface markers typically associated with unconventional T cells and innate‐lymphoid cell (ILC) subsets including the C‐type lectin CD161,^3^ IL‐18Rα (CD218)^4^ as well as the ectopeptidase CD26.^6^, ^20^ Upon activation, MAIT cells produce large quantities of proinflammatory cytokines IFNγ and TNF^6^ and under certain microenvironmental conditions, IL‐17A^5^, ^6^, ^18^, ^21^ and IL‐22.^21^ Thus, MAIT cells are poised to mount a proinflammatory peripheral response to microbial infection.

MAIT cells also appear to be perturbed in several noninfectious diseases, including autoimmunity,^22^, ^23^, ^24^, ^25^, ^26^, ^27^, ^28^ metabolic disorders^29^ and cancer,^30^, ^31^, ^32^, ^33^, ^34^ as well as viral infection.^35^, ^36^, ^37^, ^38^ With growing interest in the field of MAIT cell biology, correct identification of MAIT cells is critical to determine their role in health and disease. Typically, this has relied on the use of a monoclonal antibody (mAb) directed against the TRAV1‐2 gene segment used by MAIT cells.^3^, ^7^, ^39^ Because conventional T cells can also use the TRAV1‐2 gene segment, MAIT cells are usually also defined by their high expression of CD161,^3^ IL‐18Rα,^4^ or CD26.^6^, ^20^ However, it is unclear how reliable these surrogate markers are for identifying all subsets of MAIT cells, especially in the context of disease. For example, a study of HIV‐infected individuals suggested that MAIT cells may lose expression of CD161 upon activation,^37^ and many reports suggest a reduction in MAIT cells defined by these surrogate markers in disease settings,^27^, ^28^, ^40^, ^41^, ^42^, ^43^, ^44^ and in the context of aging.^42^, ^45^

Recently, MR1‐Ag‐loaded tetramers have been developed for the specific identification of MAIT cells.^7^, ^12^ Here, using these tetramers, we examine MAIT cells and five subsets thereof, defined by CD4, CD8α and CD8β, in healthy human peripheral blood. Our findings show that these subsets can be phenotypically and functionally distinct, and while surrogate markers generally enrich for MAIT cells, for some subsets and in some individuals, these markers fail to accurately capture all of these cells. We also show that most MAIT cell subsets vary with age and that their numbers directly correlate with Natural Killer T (NKT) cells and Vδ2^+^ γδ T cells. This study should serve as a valuable guide for the interpretation of earlier studies prior to the availability of MR1‐Ag tetramers, and the analysis and isolation of MAIT cells from human blood in health and disease.

## Results

### Innate‐like T cell frequency

**Figure 1:**
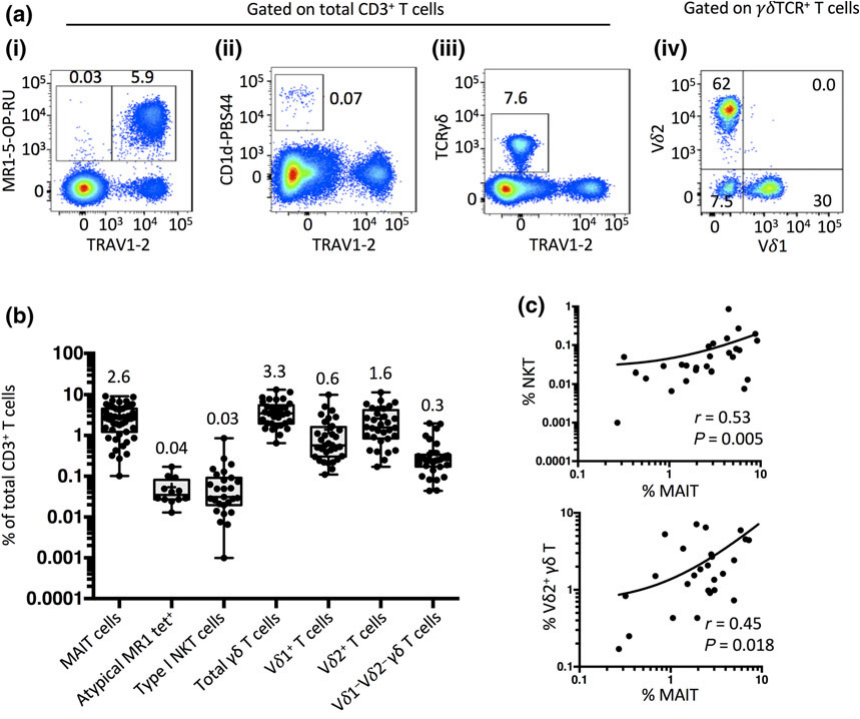
Enumeration of unconventional T‐cell subsets. **(a)** Flow cytometric plots showing example gating of (i) MAIT cells and atypical MR1 tetramer^+^ TRAV1‐2^−^ T cells, (ii) Type I NKT cells and (iii) γδ T cells, gated on total CD3^+^ T cells, and (iv) Vδ1 *versus* Vδ2 expression on γδ T cells. **(b)** Box and whisker plots showing the percentage of innate‐like T‐cell subsets of total CD3^+^ T cells. MAITs *n *=* *47; atypical MR1 tet^+^ *n *=* *12; Type I NKT cells *n *=* *27; γδ T cells *n *=* *33; derived from eight experiments. **(c)** Scatter plots showing donor‐matched percentages of (i) MAIT and NKT cells, or (ii) MAIT and Vδ2^+^ T cells as a proportion of total CD3^+^ cells (Spearman correlations (i) *r* = 0.53, *P *=* *0.005, *n *=* *27; (ii) *r* = 0.45, *P *=* *0.018, *n *=* *27).

Using MR1‐5‐OP‐RU tetramers to identify MAIT cells, we first established the frequency of MAIT cells compared to other unconventional T‐cell subsets, including Type I NKT cells and γδ T‐cell subsets, in a cohort of adult peripheral blood mononuclear cell (PBMC) blood donor samples (Figure 1a i–iii). MAIT cells accounted for a mean of 3.1% of total T cells, varying from 0.1% to 9.2% with an interquartile range (IQR) of 1.3–4.5% and median of 2.6%. As previously published,^14^ MR1‐5‐OP‐RU tetramer^+^ TRAV1‐2^−^ atypical MR1‐reactive T cells were much less frequent, with a mean frequency of 0.05%, ranging from 0.01% to 0.17%, IQR of 0.03–0.08% and median of 0.04% (Figure 1b). Nonetheless, the frequency of these cells was similar to Type I NKT cells which had a mean of 0.09% but a larger range, from less than 0.001–0.9%, IQR of 0.02–0.09 and median of 0.03%. The frequency of γδ T cells was comparable to that of MAIT cells, with a mean of 4.3%, a range of 0.7–13.3%, IQR of 2.0–5.5% and median of 3.5%. The majority of γδ T cells were either Vδ1^+^ or Vδ2^+^ (Figure 1a iv) accounting for a mean of 1.4 and 2.6% of total T cells, respectively (Figure 1b). Thus, MAIT cells were similar in frequency to Vδ2^+^ γδ T cells, and far more abundant than Type 1 NKT cells and atypical MR1‐5‐OP‐RU tetramer^+^ TRAV1‐2^−^ cells, which were similar to each other. Intriguingly, when comparing these populations, we found a positive correlation between the proportion of Type I NKT cells and MAIT cells (Spearman correlation *r* = 0.53, *P *=* *0.005) and Vδ2^+^ γδ T cells and MAIT cells (Spearman correlation *r* = 0.45, *P *=* *0.018; Figure 1c). In contrast, no significant correlations were observed between MAIT cells and other γδ T‐cell subsets, or with atypical TRAV1‐2^−^ MR1‐tetramer^+^ T cells and MAIT cells. This suggests that MAIT, NKT and Vδ2^+^ γδ T‐cell frequencies are controlled by similar genetic and/or environmental factors.

### MAIT cell co‐receptor distribution

**Figure 2:**
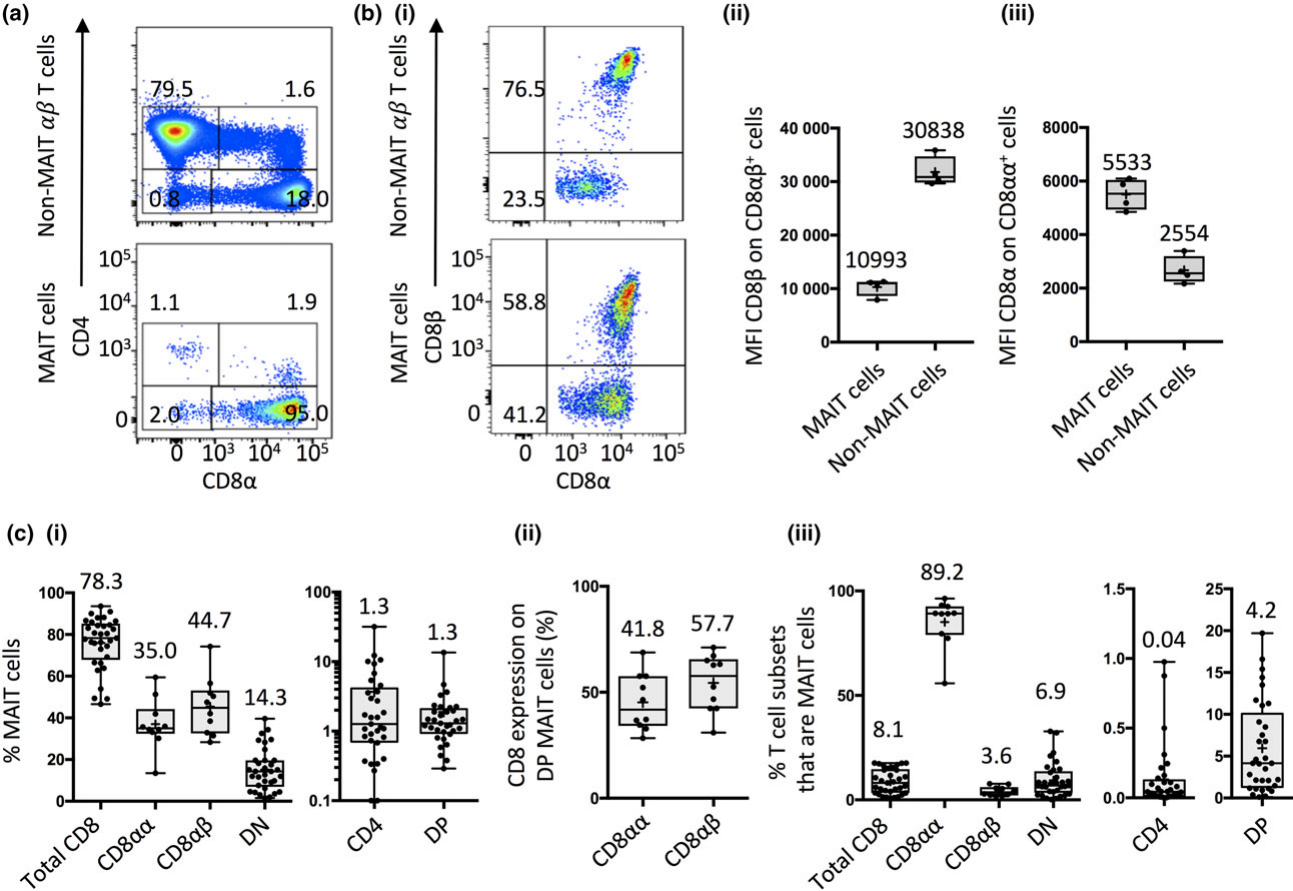
Co‐receptor distribution on MAIT cells. Flow cytometric plots showing example gating of CD4 and CD8α expression **(a)** and CD8α and CD8β expression (**b** i) on non‐MAIT αβ T cells (upper panels) and MAIT cells (lower panels). Box and whisker plots showing CD8β (**b** ii) and CD8α (**b** iii) expression on CD8αβ^+^, cells on MAIT cells and non‐MAIT αβ T cells (*n *=* *4, from two experiments). CD8β gate set based on fluorescence minus one (FMO) control. **(c)** Box and whisker plots showing: (i) the percentage of MAIT cells expressing each co‐receptor; (ii) the percentage of CD4^+^CD8^+^ DP MAIT cells that are CD8αα^+^ or CD8αβ^+^, and; (iii) the percentage of total T cells expressing each co‐receptor that are MAIT cells (total CD8^+^, DN, CD4^+^ and DP *n *=* *33; CD8αα^+^ and CD8αβ^+^ *n *=* *10; from eight experiments).

We next examined CD4 and CD8 co‐receptor expression by MAIT cells defined by MR1‐5‐OP‐RU tetramer staining. Prior to this, however, we determined the ability to co‐stain for CD8α and CD8β on MAIT cells. Staining with one or both mAb did not alter the frequency of MAIT cells identified as CD8α^+^ or CD8β^+^, although there was a slight reduction in the MFI for these markers when they were co‐labeled (Supplementary figure 1). Consistent with previous reports,^17^ the majority of human MAIT cells were CD8α^+^, and within this population most were CD8αβ^+^ (Figure 2a i, 2b i). While the CD8αβ^+^ MAIT cells expressed similar levels of CD8α compared to conventional αβ T cells, they expressed substantially lower levels of CD8β (Figure 2b i), suggesting that they likely co‐express CD8αα homodimers and low levels of CD8αβ heterodimers on their surface. Conversely, CD8αα^+^ MAIT cells expressed higher levels of CD8α than other non‐MAIT CD8αα^+^ αβ T cells (Figure 2b ii–iii). Control samples that were stained with either MR1‐5‐OP‐RU tetramers or control MR1‐Ac‐6‐FP tetramers^46^ confirmed that each subset was specifically stained with MR1‐5‐OP‐RU tetramers (Supplementary figure 2). As a proportion of total MAIT cells, a median of 35.0% and range of 13.5–59.5% were CD8αα^+^, whereas a median of 44.7% expressed CD8αβ heterodimers, ranging from 28.3% to 74.2% (Figure 2c). A median of 14.3% of MR1‐5‐OP‐RU tetramer^+^ MAIT cells were DN, ranging from 1.6% to 39.6%, while a minor proportion (median 1.3%) of these cells were CD4^+^CD8^−^ single positive (SP; hereafter referred to as CD4^+^ MAIT cells) or CD4^+^CD8^+^ DP, (median 1.3%) (Figure 2a i, 2c). Closer analysis of the DP MAIT cells revealed that the distribution of CD8αα and CD8αβ was similar to that of CD4^−^CD8α^+^ MAIT cells with medians of 41.8% (range 28.4–68.8%) and 57.7% (range 31.2–71.6%), respectively (Figure 2c ii). Also, similar to CD4^−^CD8αβ^+^ MAIT cells, the CD4^+^CD8αβ^+^ MAIT cells had a lower CD8β MFI, suggesting co‐expression of both CD8αα homodimers and low levels of CD8αβ heterodimers (data not shown).

As a percentage of T‐cell subsets, MAIT cells made up medians of 8.1 and 6.9% of CD8^+^ T cells and DN T cells, ranging between 0.7% and 17.6% and 0.2 and 32.8%, respectively. MAIT cells accounted for a median of 89.2% of total CD8αα^+^ cells, varying from 55.8% to 96.4%, while of total CD8αβ^+^ T cells they were 3.6% and ranged from 1.7% to 7.7%. In contrast, MAIT cells only made up a minor proportion of total CD4^+^ and DP T cells with medians of 0.04 and 4.2% and ranges of 0–1.0% and 0.1–19.7%, respectively (Figure 2c iii).

Taken together, while in most individuals the majority of MAIT cells were CD8αβ^+^, there was substantial inter‐individual variation in co‐receptor distribution, including some extreme outliers. For example, in one individual (G73), 31.8% of MAIT cells were CD4^+^ (Supplementary figure 3), most of which were intermediate to negative for CD161 expression.

### MAIT cell subsets differentially decline with age

**Figure 3:**
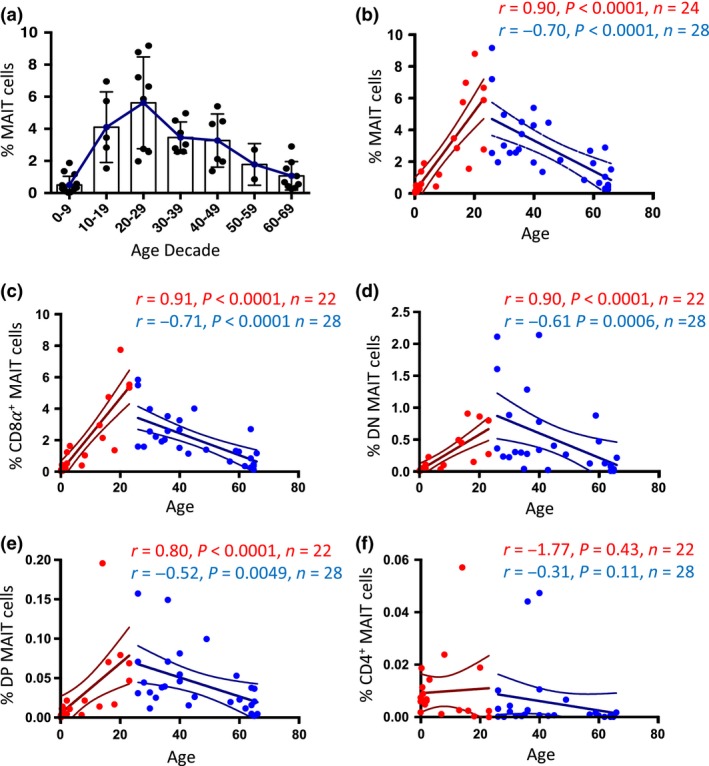
Selective decrease in MAIT cell subsets with age. **(a)** Box plot showing MAIT cell frequency of total CD3^+^ T cells. **(b–f)** Scatter plots showing donor age *versus* the proportion of total CD3^+^ T cells that are: **(b)** MAIT cells **(c)** CD8*α* ^+^ MAIT cells **(d)** DN MAIT cells **(e)** DP MAIT cells or **(f)** CD4^+^ MAIT cells. Red data points = ages 0–25; blue data points = ages 26–70. Statistical tests are Spearman correlations. Linear regression lines and 95% confidence intervals are also depicted. Samples from young donors (ages 0–14) had been partially analyzed in a previous study,^16^ but without full CD4/CD8 subset analysis as depicted here.

Next, we examined how MAIT cell subsets defined by CD4 and CD8 expression change with age ranging from birth to 70 years old. The under‐20‐years‐old donor samples have been depicted in a previous study^16^ but without the detailed subset analysis shown here. Consistent with previous reports that focused on CD8^+^ MAIT cells using surrogate markers to identify these cells,^42^, ^45^ we confirmed that total MAIT cells increase in proportion over the first three decades of life, peaking between the ages of 20–29, before gradually declining in the decades thereafter (Figure 3a). Statistical analysis showed a strong positive correlation between MAIT cell frequency and age between birth and 25 years of age (correlation *r* = 0.90, *P *<* *0.0001; linear regression *r* ^2^ = 0.70, *P *<* *0.0001) and a strong negative correlation between 25 and 70 years of age (correlation *r* = −0.70, *P *<* *0.0001; linear regression *r* ^2^ = 0.46, *P *<* *0.0001; Figure 3b). Analysis of MAIT cell subsets defined by CD4 and CD8 co‐receptor expression suggested that the initial increase in MAIT cell proportions correlated most strongly for CD8α^+^ MAIT cells (correlation *r* = 0.91, *P *<* *0.0001; linear regression *r* ^2^ = 0.77, *P *<* *0.0001), followed by DN MAIT cells (correlation *r* = 0.90, *P *<* *0.0001; linear regression *r* ^2^ = 0.63, *P *<* *0.0001) and DP MAIT cells (correlation *r* = 0.80, *P *<* *0.0001 linear regression *r* ^2^ = 0.35, *P *=* *0.035), whereas the subsequent decline in MAIT cell proportions correlated most strongly in the CD8α^+^ MAIT cells (correlation *r* = −0.71, *P *<* *0.0001; linear regression *r* ^2^ = 0.47, *P *<* *0.0001) followed by DN MAIT cells (correlation *r* = −0.61, *P *=* *0.0006; linear regression *r* ^2^ = 0.22, *P *=* *0.01), and DP MAIT cells (correlation *r* = −0.52, *P *=* *0.0049; linear regression *r* ^2^ = 0.21, *P *=* *0.0135; Figure 3c–e). Of note, no correlation was observed for CD4^+^ MAIT cell proportions for either the young or old age ranges (Figure 3f). These results suggest that MAIT cell subsets may be differentially regulated and should be included in analysis where possible. They also highlight that appropriate age‐matching of patient cohorts and healthy controls is critical to analyzing MAIT cells in disease.

### Comparison of MR1‐Ag tetramers and surrogate phenotyping techniques

**Figure 4:**
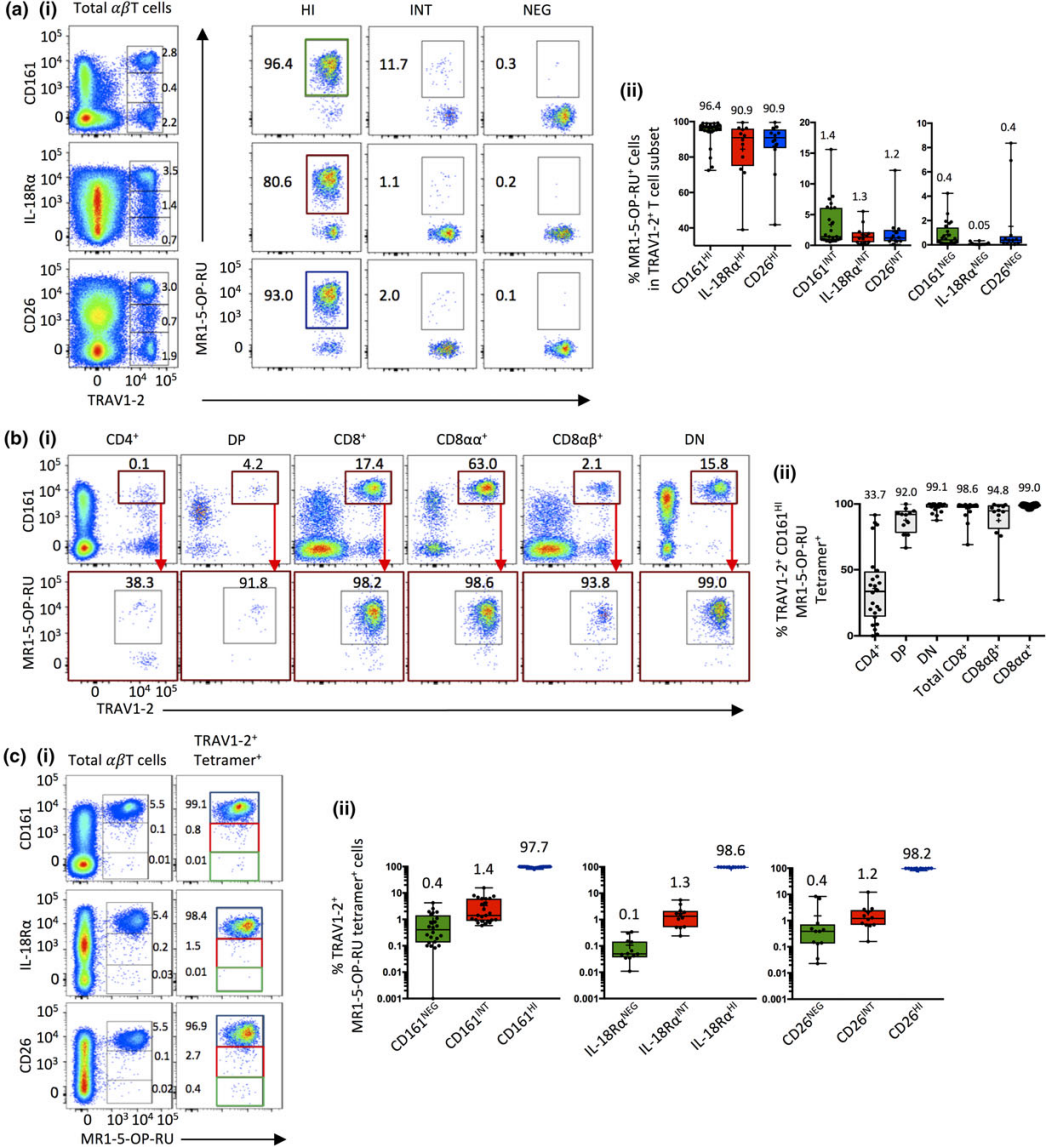
Comparison of MR1‐Ag tetramers and surrogate phenotyping techniques. **(a)** (i) Representative flow cytometric pseudo‐color plots showing example gating of MAIT cells using anti‐TRAV1‐2 mAb *versus* CD161 in one cocktail, or IL‐18Rα or CD26 in another cocktail on total T cells, followed by MR1‐5‐OP‐RU tetramer staining on TRAV1‐2^+^ CD161/IL‐18Rα/CD26 high, intermediate or negative cells. (ii) Box and whisker plots showing percentage TRAV1‐2^+^, CD161 (green; *n *=* *24), IL‐18Rα (red; *n *=* *12) and CD26 (blue; *n *=* *12) High (HI), Intermediate (INT) and Low (LOW) cells that are MR1‐5‐OP‐RU tetramer^+,^ derived from three experiments. **(b)** (i) Representative flow cytometric pseudo‐color plots showing example gating of MAIT cells using anti‐TRAV1‐2 mAb *versus* CD161 for T cell co‐receptor subsets (upper panel) and MR1‐5‐OP‐RU tetramer staining on TRAV1‐2^+^ CD161^HI^ cells for each co‐receptor (lower panel). (ii) Box and whisker plots showing percentage TRAV1‐2^+^, CD161^HI^ cells that are MR1‐5‐OP‐RU tetramer^+^ for each co‐receptor (CD4^+^, DN and CD8^+^ *n *=* *24; DP, CD8αα^+^ and CD8αβ^+^ *n *=* *12, from three experiments). **(c)** (i) Representative flow cytometric pseudo‐color plots showing example gating of MAIT cells using MR1‐5‐OP‐RU tetramer *versus* CD161, IL‐18Rα or CD26 on total T cells (left panels) or MR1‐5‐OP‐RU tetramer^+^ TRAV1‐2^+^ T cells (right panel). (ii) Box and whisker plots showing percentage TRAV1‐2^+^ MR1‐5‐OP‐RU tetramer^+^ cells that are HI (blue), INT (Red) or NEG (green) for CD161 (*n *=* *24), IL‐18Rα (*n *=* *12) and CD26 (*n *=* *12), derived from three experiments.

MAIT cells exhibit a unique cell surface phenotype, including expression of high levels of the C‐type lectin CD161,^3^ the IL‐18Rα chain CD218^4^ as well as the ectopeptidase CD26.^6^, ^20^ Indeed, these markers are often used in combination with a mAb directed against the TRAV1‐2 TCR variable domain to identify and study MAIT cells. However, it is unclear how precisely these markers define MAIT cells, whether all MAIT cells express them and whether all T cells identified by these markers are MAIT cells. We therefore investigated how accurate these markers are for identifying MR1‐5‐OP‐RU tetramer^+^ TRAV1‐2^+^ MAIT cells in healthy donors. We first determined how many MR1‐5‐OP‐RU tetramer^+^ cells are in the TRAV1‐2^+^ cell subsets as separately defined by CD161, IL‐18Rα and CD26 expression (Figure 4a). Medians of 96.4% (CD161) and 90.9% (for each of IL‐18Rα and CD26) of cells expressing high levels of these markers, respectively, co‐labeled with MR1‐5‐OP‐RU tetramers. However, for each of these markers, there was inter‐donor variability, with up to 27.5, 61.0 and 58.2% of TRAV1‐2^+^ CD161^HI^, CD218^HI^ and CD26^HI^ cells, respectively failing to label with MR1‐5‐OP‐RU tetramers in some individuals. Furthermore, some MR1‐5‐OP‐RU tetramer^+^ MAIT cells were detected within the populations of TRAV1‐2^+^ cells that expressed intermediate or negative levels of these markers (Figure 4a ii).

To further investigate the value of surrogate markers to identify MAIT cell subsets, TRAV1‐2^+^ CD161^HI^ cells, representing the most accurate of the surrogate marker combinations, were gated into subpopulations based on CD4/CD8 co‐receptor usage (Figure 4b). While MAIT cells were detected in each subpopulation, the effectiveness of these markers for identifying CD4^+^ MAIT cells was highly inaccurate, with a median of only 33.7% (IQR of 15.1–48.3) of CD4^+^ TRAV1‐2^+^ CD161^HI^ cells labeling with MR1‐5‐OP‐RU tetramer. This was further exemplified by performing single cell TCR sequencing on CD4^+^ CD161^+^ cells from one donor. Cells at the upper most edge of the CD161^HI^ CD4^+^ MAIT gate *versus* the lower edge of this gate showed that while the cells with highest CD161 expression expressed the canonical MAIT TCR‐α chain, the cells at the lower edge of the CD161^+^ cells expressed both canonical MAIT and diverse non‐MAIT TCR‐α chains, supporting the MR1 tetramer data showing that this population does not reliably represent MAIT cells (Supplementary figure 4). Upon examination of other subsets of CD161^HI^ TRAV1‐2^+^ cells; DP, CD8αβ^+^, DN and CD8αα^+^ T cells showed medians of 92.0% (IQR 78.5–94.4%) 94.8%, (IQR 81.5–99.7), 99.1% (IQR 97.8–99.8) and 99.0% (IQR 98.8–99.6) of MR1‐5‐OP‐RU tetramer^+^ cells, respectively. Taken together, while the CD161^HI^ TRAV1‐2^+^ phenotype is a reasonably accurate indicator of CD8αα^+^ and DN MAIT cells, for other MAIT cell populations (CD4^+^, DP and CD8αβ^+^) this approach is not very reliable.

Next, expression of the commonly used surrogate markers on total TRAV1‐2^+^ MR1‐5‐OP‐RU tetramer^+^ MAIT cells was determined (Figure 4c). As expected, the majority of MAIT cells expressed high levels of CD161, IL‐18Rα and CD26 (medians of 97.7, 98.6 and 98.2%, respectively). Nonetheless, a small proportion of MAIT cells expressed low or intermediate levels of these markers (medians of 0.4 and 1.4%, respectively for CD161; 0.1 and 1.3%, respectively for IL‐18Rα; 0.4 and 1.2%, respectively for CD26) (Figure 4c ii). Analysis of co‐expression of CD26 and CD161 on MAIT cells from four donors suggested that minor populations of each of CD161^−^CD26^+^, CD161^+^CD26^−^ and CD161^−^CD26^−^ exist, with CD26^−^CD161^+^ being the most prominent of the three populations (Supplementary figure 5a). From two of these donors, we detected a clear subpopulation of TRAV1‐2^+^ MR1‐5‐OP‐RU tetramer^+^ MAIT cells that were negative for CD26, and in one donor these cells expressed lower levels of CD161 compared to the rest of the MAIT population, as well as being CD27^−^CD28^−^Tbet^−^ (Supplementary figure 5a, donor D3 and Supplementary figure 5b). This highlights that while in most cases, the surrogate markers, particularly CD161, accurately identify most MAIT cells, not all MAIT cells are identified with this approach, and in some outlying individuals these markers can be highly inaccurate. Thus, while the combination of CD161 and TRAV1‐2 identifies the great majority of MAIT cells, not all CD161^+^ T cells are MAIT cells and not all MAIT cells are identified with these markers.

### MAIT cell surface phenotype

**Figure 5:**
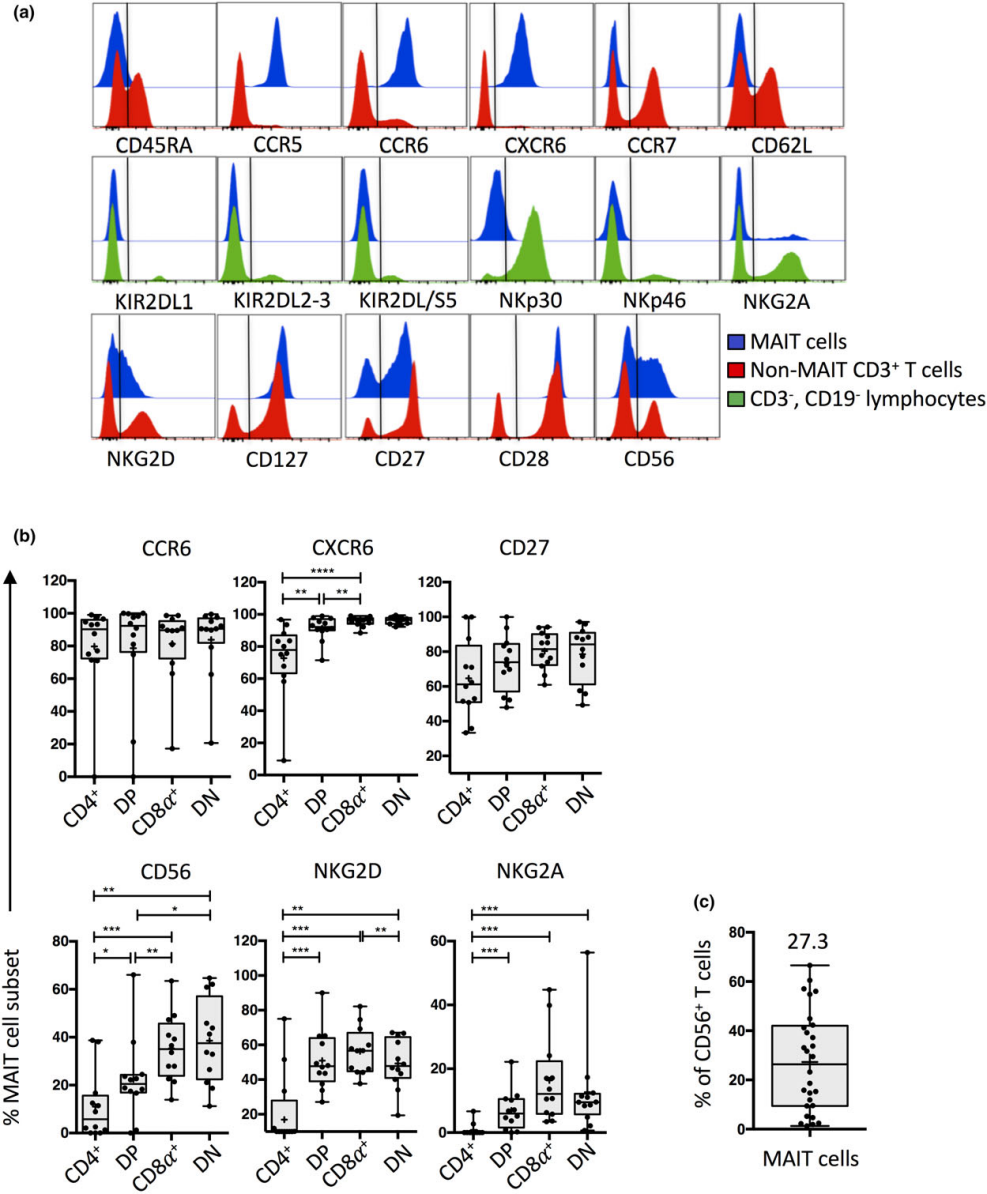
Surface phenotyping of MAIT cells. **(a)** Representative histogram overlays of surface marker expression on MAIT cells (blue histograms), non‐MAIT CD3^+^ T cells (red histograms) and CD3^−^, CD19^−^ lymphocytes (green histograms) **(b)** Box and whisker plots showing the percentage of MAIT cell subsets expressing CCR6, CXCR6, CD27, CD56, NKG2D and NKG2A (*n *=* *12 from two separate experiments). MAIT cell subsets were gated as per Figure 2a after initially gating on CD3^+^TRAV1‐2^+^MR1‐5‐OP‐RU tetramer^+^, viable lymphocytes. The statistical test was a Wilcoxon matched‐pairs signed‐rank test. **(c)** Box and whisker plot showing the percentage of total CD56^+^ T cells that are MAIT cells (*n *=* *28, from three experiments).

Next, phenotypic analysis of TRAV1‐2^+^ MR1‐5‐OP‐RU tetramer^+^ MAIT cells was performed for other cell surface markers of interest, comparing them to other T cells or other CD3^−^ CD19^−^ lymphocytes which includes NK cells (Figure 5a). These data showed that the vast majority of MAIT cells were CD45RA^−^, CCR5^+^, CCR6^+^, CXCR6^+^, CCR7^−^ and CD62L^−^, suggesting that MAIT cells from blood are Ag‐experienced tissue‐homing cells. MAIT cells were also negative for killer‐cell immunoglobulin‐like receptors (KIR) including KIR2DL1, KIR2DL2, KIR2DL3, KIR2DL5 and KIR2DS5 as well as the natural cytotoxicity receptor (NCR) NKp46, with at best only a minor subset expressing low levels of NKp30, whereas they were heterogeneous for NKG2A and NKG2D (Figure 5a). MAIT cells typically expressed high levels of the co‐stimulatory molecule CD28 and the IL‐7Rα chain CD127, whereas they expressed variable levels of the costimulatory molecule CD27, and the adhesion molecule CD56. We also noted that the NKG2D^+^ MAIT cells express low‐intermediate levels of NKG2D compared to conventional T cells which were either low or high for this marker.

We next determined whether markers with heterogeneous expression on total MAIT cells varied between CD4^+^, DP, CD8α^+^ and DN MAIT cell subsets (Figure 5b). CCR6 and CD27 expression were similar between subsets, whereas CXCR6, CD56, were expressed at lower levels on CD4^+^ and DP MAIT cells compared to other MAIT cell subsets. NKG2D and NKG2A were also differentially expressed between subsets, with CD4^+^ MAIT cells expressing the lowest levels of these markers and minor differences in NKG2D expression were also observed between the prominent CD8α^+^ and DN subsets, as previously reported.^47^ Thus, in general the CD4^+^ MAIT cell subset appeared to be the most distinct, exhibiting significantly lower proportions of cells expressing each of CXCR6, CD56, NKG2D and NKG2A compared to the other MAIT cell subsets.

CD56 was of particular interest because this marker is typically associated with NK cells and innate‐like T cells, and many studies have incorrectly classified CD56^+^ T cells as NKT cells.^48^ Here, we determined that many CD56^+^ cells co‐labeled with MR1‐5‐OP‐RU tetramers although there was high inter‐donor variability (mean of 27.3%, ranging from 1.3 to 66.6%; Figure 5c). This indicates that while CD56 is a poor surrogate marker of MAIT cells, studies that have examined the function of CD56^+^ T cells may have actually included MAIT cells in their analysis.

### MAIT cell transcription factor profile

**Figure 6:**
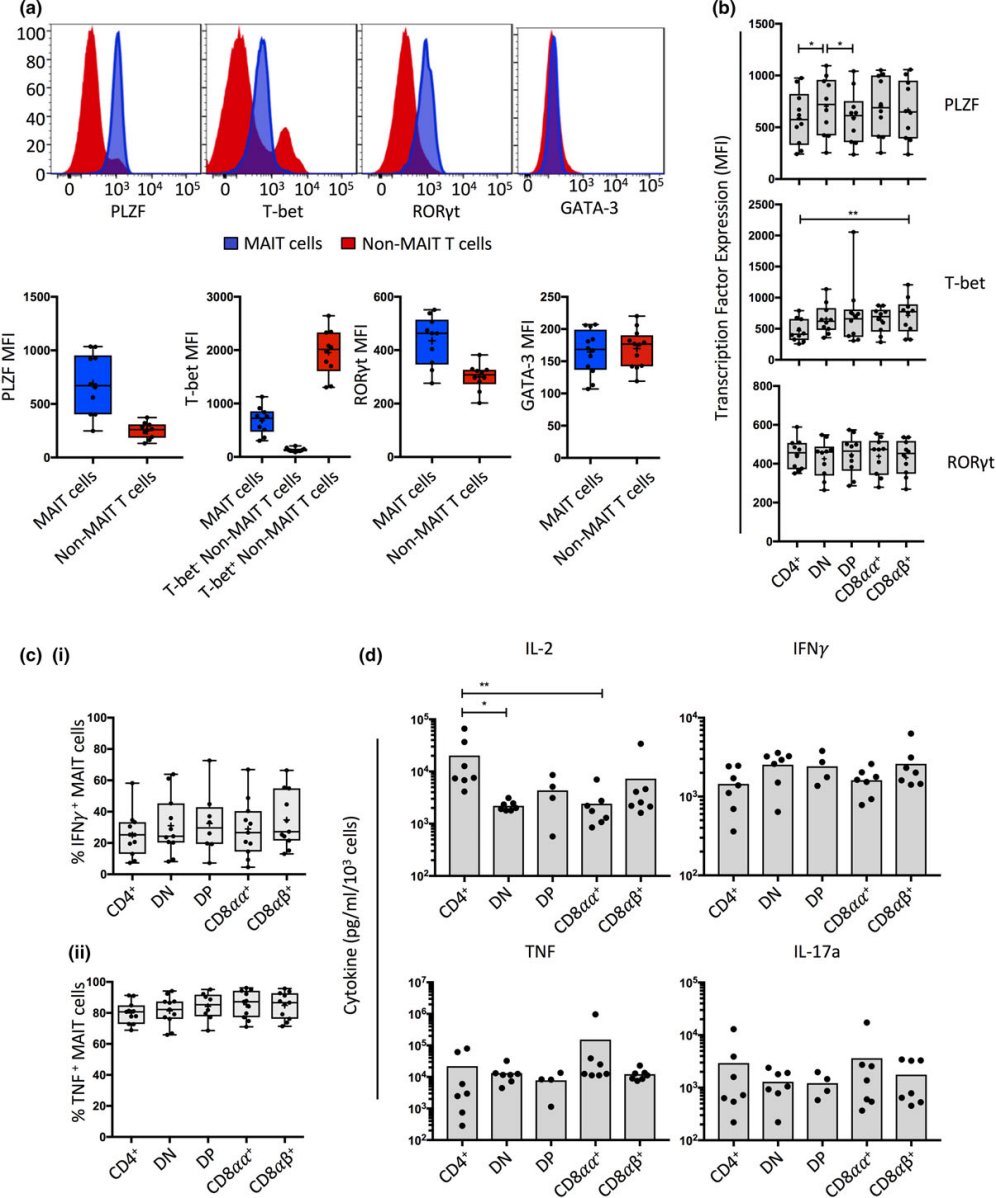
Transcription factor and cytokine profile of MAIT cell subsets. **(a)** Upper panel: Representative flow cytometric histograms showing transcription factor staining on MAIT cells (blue histograms) overlaid above total non‐MAIT αβ T cells (red histograms). Lower panel: Box and whisker plots showing the MFI of transcription factor staining on MAIT cells (blue boxes) and total non‐MAIT T cells (red boxes) for PLZF, RORγt, T‐bet and GATA‐3. (*n *=* *12 from three experiments). **(b)** Box and whisker plots showing the MFI of transcription factor staining between MAIT cell subsets for PLZF, T‐bet and RORγt. (*n *=* *10 from two separate experiments). **(c)** Box and whisker plots showing the percentage of IFNγ^+^ or TNF^+^ MAIT cells subsets after 7 h stimulation of donor PBMCs with PMA and ionomycin. (*n *=* *11, from three experiments). **(d)** Box plots showing supernatant cytokine levels after FACS‐sorted MAIT cell subsets were stimulated for 24 h with PMA and ionomycin. (*n *=* *7 for CD4^+^, DN, CD8αα^+^ and CD8αβ^+^ MAIT cells, and *n *=* *4 for DP MAIT cells from two separate experiments). Statistical analyses in b‐d were performed using Friedman tests with Dunn's multiple comparison *post hoc* tests.

We next performed intracellular transcription factor staining with a panel of mAbs directed against key master transcriptional regulator proteins (Figure 6). MAIT cells were positive for the innate transcription factor PLZF and the T_H_17 master regulator RORγt, in line with previous reports.^3^, ^14^, ^17^, ^18^, ^49^ Interestingly, MAIT cells expressed intermediate levels of T_H_1 transcription factor T‐bet rather than high levels that characterized a subset of non‐MAIT T cells, and they did not express the T_H_2 transcription factor GATA‐3 (Figure 6a).^18^ Of note however, one donor had a subpopulation of T‐bet^hi^ MAIT cells (Supplementary figure 6b) again highlighting the diversity of these cells between and within individuals. The subsets defined by CD4 and CD8 were largely similar for transcription factor expression, although CD4^+^ and DP MAIT cells expressed moderately lower levels of PLZF than the DN subset, and CD4^+^ MAITs expressed lower levels of T‐bet than the CD8αβ subsets (Figure 6b). Thus, despite some differences in the cell surface phenotypes, we show that all subsets of MR1 tetramer^+^ MAIT cells defined by CD4 and CD8 expression exhibit an innate‐like (PLZF^+^), T_H_1 (T‐bet^+^), T_H_17 (RORγt^+^) transcription factor profile, albeit with some variation between subsets and between individuals.

### MAIT cell subset cytokine production

To determine whether a similar proportion of MAIT cells in each subset could produce cytokines, healthy PBMCs were stimulated *in vitro* with phorbol 12‐myristate 13‐acetate (PMA) and ionomycin for 7 h prior to intracellular cytokine staining for IFNγ and TNF (Figure 6c). No significant differences in ability to produce TNF or IFNγ were observed between any of these subsets. In order to examine a broader array of cytokines, MAIT cells were purified by magnetically enriching MR1‐5‐OP‐RU tetramer^+^ cells from blood packs, and the CD4/CD8‐defined subsets purified by flow cytometric cell sorting (Supplementary figure 6). Purified cells were stimulated *in vitro* with PMA and ionomycin for 24 h at which point culture supernatants were analyzed for the presence of cytokines: IFNγ, TNF, IL‐2, ‐4, ‐5, ‐10, ‐13, ‐17A (Figure 6d). The cytokine response was characterized by IL‐2, IFNγ, TNF and IL‐17A production. MAIT cell subsets produced similar quantities of most cytokines, with the exception of CD4^+^ MAIT cells which produced considerably higher (>5 fold) levels of IL‐2 than both DN and CD8αα^+^ MAIT cells. Type 2 cytokines, including IL‐4, ‐5 and ‐13, as well as the immunosuppressive cytokine IL‐10, were not detected by any subpopulation of MAIT cells (data not shown). Thus, MAIT cell subsets, as defined by co‐receptor expression, appear to have similar cytokine profiles in response to mitogenic stimulation, with a notable exception of CD4^+^ MAIT cells producing higher quantities of IL‐2.

### MAIT cell TRAJ‐gene usage

**Figure 7:**
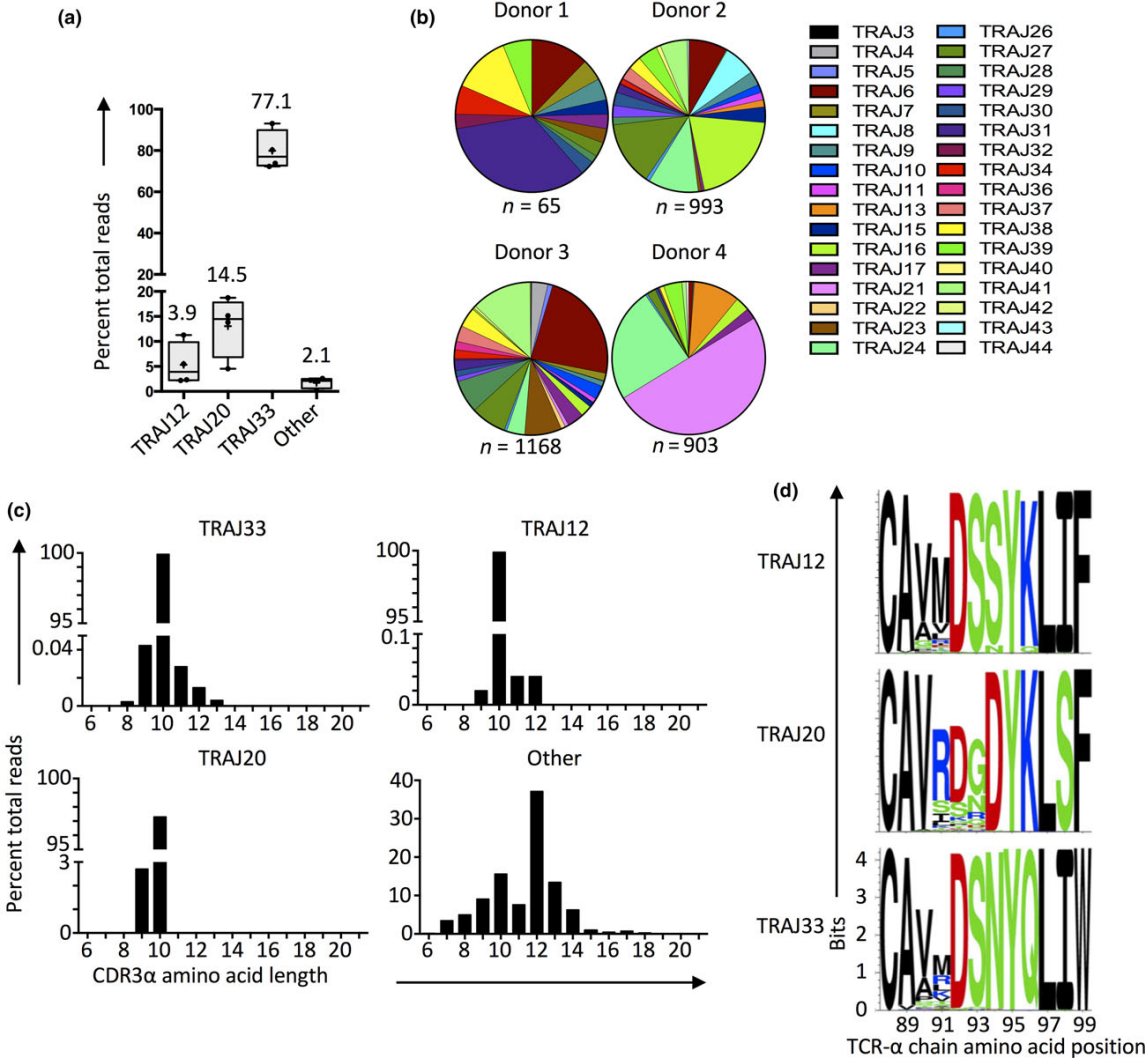
TRAJ gene usage by TRAV1‐2^+^ MAIT cells. **(a)** Box and whisker plots showing percent total productive TRAV1‐2^+^ reads recombined with TRAJ12, TRAJ20, TRAJ33 or other TRAJ genes in amplified cDNA from MR1‐5‐OP‐RU tetramer^+^ TRAV1‐2^+^ T cells subjected to deep sequencing. Data are from 4 human donors (*n *=* *43 969, 45 454, 46 126 and 46 553 functional TRAV1‐2^+^ reads for donors 1–4, respectively). **(b)** Pie charts showing distribution of TRAJ genes by noncanonical TRAV1‐2^+^ TCR‐α chain transcripts from four individual donors. **(c)** Bar graphs showing CDR3‐α junction amino acid length distribution for TRAJ12^+^, TRAJ20^+^, TRAJ33^+^ and Other TRAV1‐2^+^ TCR‐α chain transcripts pooled from four individual donors (derived from *n *=* *9513, 19 522, 141 853 and 3006 transcripts, respectively). **(d)** Sequence logos depicting the amino acid distribution at the CDR3‐α junction in TRAJ12^+^, TRAJ20^+^ or TRAJ33^+^ TCR‐α chain transcripts that are 10 amino acids in junctional length (derived from *n *=* *9451, 18 910 and 141 274 transcripts, respectively).

MAIT cells are defined by expression of a semi‐invariant TCR that utilizes an almost invariant TCR‐α chain (pairing TRAV1‐2 with TRAJ33) that drives recognition of riboflavin‐derived Ags presented by MR1.^50^, ^51^ MR1 tetramers have been used to confirm that while this is true for the majority of MAIT cells, TRAJ12 and TRAJ20 can also be incorporated into the TRAV1‐2^+^ MAIT cell repertoire,^7^ with conserved use of a TRAJ‐gene‐encoded tyrosine residue at position 95 (Tyr95α), providing the molecular basis for this gene usage.^46^ Moreover, a recent report suggests that the MAIT TCR‐α chain repertoire may reflect distinct tissue‐tropic subsets within individuals,^10^ and may also extend beyond the use of TRAJ33, TRAJ12 and TRAJ20 to include TRAJ genes that do not encode a Tyr95α.^52^, ^53^ Thus, variation within the MAIT TCR‐α chain repertoire are another potential means for MAIT cell subset diversity. TRAJ‐gene variation has been shown using surrogate marker‐based MAIT cell identification methods at both a single cell and deep sequencing level.^7^, ^10^, ^20^, ^53^ However, MR1‐Ag tetramer‐based repertoire studies have so far been limited to single cell TCR sequencing on less than 200 MAIT cell clones.^7^ Given our findings above (Figure 4) that some T cells defined by surrogate phenotypes are not MAIT cells, it was important to further examine the frequency and TCR‐α chain repertoire of MAIT cells defined by MR1‐5‐OP‐RU tetramer using TCR‐α chain deep sequencing. For this purpose, bulk populations of FACS‐sorted TRAV1‐2^+^ MR1‐5‐OP‐RU tetramer^+^ MAIT cells from four healthy donors were examined (Figure 7). With these data, we confirm that the majority of MAIT cells utilized a TCR‐α chain composed of TRAV1‐2 joined to TRAJ33 (median 77.1%), but many alternately used TRAJ12 or TRAJ20 (medians of 3.9 and 14.5%, respectively) (Figure 7a). Each donor also had a small number of transcripts that used noncanonical TRAJ genes not encoding Tyr95α joined to TRAV1‐2 (median 2.1%). The noncanonical TRAJ genes were diverse, with no apparent conservation between donors (Figure 7b), and while the TRAJ12^+^, TRAJ20^+^ and TRAJ33^+^ MAIT TCR‐α chains had a highly biased CDR3α junction amino acid length of 10 amino acids, the rare noncanonical TCR‐α chains were much more variable (Figure 7c). In line with previous reports,^7^, ^9^ detailed analysis of the canonical MAIT TCR‐α chains highlighted that they are largely germline encoded, although some sequence variation was permitted at positions 90‐91α for TRAJ12^+^ and TRAJ33^+^ transcripts and 91‐93α for TRAJ20^+^ transcripts (Figure 7d). Importantly, Tyr95α was highly conserved in all three groups of TCR‐α chain (Figure 7d). Accordingly, the TRAV1‐2^+^ MAIT TCR‐α chain repertoire is highly biased toward largely germline encoded canonical sequences with a CDR3α junctional length usually limited to 10 amino acids. Moreover, noncanonical TRAJ‐gene usage is present, albeit uncommon, within the TRAV1‐2^+^ MR1‐5‐OP‐RU tetramer^+^ MAIT cell population.

Previous structural studies have illustrated that the Tyr95α residue plays a conserved molecular role in TRAJ33^+^ and TRAJ20^+^ MAIT TCR binding to MR1‐5‐OP‐RU complexes, but that the network of interactions between the TCR‐α and TCR‐β chains are different between the two.^46^ To gain insight into the biological significance of this, TCR constructs were prepared which encoded a TRAJ33^+^ (clone M33‐64), TRAJ12^+^ (clone M12.64) or TRAJ20^+^ (clone M20‐64) MAIT TCR, as well as a negative control TRAJ4^+^ TCR that is TRAV1‐2^+^, TRBV6‐4^+^, but does not recognize MR1‐5‐OP‐RU (clone M4‐64). These TCR sequences have been previously published.^7^ All 4 TCRs utilized TRBV6‐4 and thus only differed in CDR3α/TRAJ and CDR3β/TRBJ sequence. Mutant TCR constructs were also prepared which all expressed the same CDR3β/TRBJ sequence as the M33‐64 TCR such that the only differences between the mutant TCRs was the CDR3α/TRAJ sequence (M12.64 β‐mutant, M20‐64 β‐ mutant and M4‐64 β‐mutant). HEK293T cells were then separately transfected to express each of the wildtype and β‐mutant TCRs, and subsequently stained with MR1‐5‐OP‐RU tetramers (Supplementary figure 7a). As expected, the 3 MAIT TCRs stained brightly with MR1‐5‐OP‐RU tetramers whereas the M4‐64 TCR did not. Strikingly, the two MAIT TCR β‐mutants exhibited a loss of 60–65% of staining intensity compared to their wildtype counterparts (Supplementary figure 7a, b). Moreover, expression of the M33‐64 TCR‐β chain did not permit the M4‐64 TCR to recognize MR1‐5‐OP‐RU complexes as shown in the M4‐64 β‐mutant line. These data suggest that TRAJ33, TRAJ12 and TRAJ20^+^ MAIT TCRs have discrete CDR3β repertoires that complement the TRAJ/CDR3α composition. Thus, while the TRAJ gene usage does not appear to directly modulate recognition of 5‐OP‐RU, it may provide an indirect mechanism for establishing a broader CDR3β repertoire by the MAIT cell population, possibly allowing for discrimination of other antigens via CDR3β, such as folate derivative 6‐FP.^14^

## Discussion

Because of their high frequency, proinflammatory capacity and potent antimicrobial activity, there is great interest in MAIT cell biology from both basic immunology and clinical research perspectives. Typically, human MAIT cells have been identified using flow cytometry by combining anti‐TRAV1‐2 with anti‐CD161, IL‐18Rα or CD26. Given that these molecules can be modulated on T cells, it was unclear how specific and accurate these surrogate markers were for identifying MAIT cells in health and disease. Another method used to define MAIT cells is an MR1‐mediated functional response, which can help to show that a population contains MR1‐Ag reactive cells; however, this is not ideal for determining if all cells in a population are MAIT cells, nor is it likely to detect a small subset of MAIT cells in a population. MR1‐5‐OP‐RU tetramers now permit the identification of MAIT cells based on their MR1‐restricted TCR specificity. Because many clinical studies have used surrogate phenotyping techniques, and often only focus on CD8^+^ MAIT cells in their analysis,^23^, ^24^, ^29^, ^30^, ^36^, ^40^, ^41^, ^44^, ^54^, ^55^, ^56^, ^57^, ^58^, ^59^ we felt it was important to determine how well these approaches compare to the use of MR1 tetramers to examine all MAIT cell populations. Here, we analyzed a large cohort of healthy human PBMC samples to both establish the phenotypic characteristics of human MAIT cells, and subsets thereof, using MR1‐5‐OP‐RU tetramers, as well as to establish a comparison of the use of MR1‐5‐OP‐RU tetramers with surrogate mAb‐based identification techniques.

Our data suggest that identification of DN and CD8^+^ MAIT cells using TRAV1‐2 and CD161, IL‐18Rα or CD26 mAbs is generally consistent with results derived from MR1 tetramer staining, at least in healthy donors. However, we have observed inter‐individual variability in how tightly these surrogate markers correlate with MR1 tetramer staining. It is also possible that some of these markers are modulated in disease states. In a study of MAIT cells in HIV patients, Leeansyah *et al*.^37^ showed that the TRAV1‐2^+^ CD161^HI^ population decreased with disease progression, and noted a reciprocal increase in the TRAV1‐2^+^ CD161^−^ population, and an accumulation of cells with a MAIT‐like phenotype including CD8α expression, biased TCR β‐chain usage, as well as PLZF expression.^37^ While another study using MR1 tetramers did not observe this downregulation of CD161,^38^ this may be explained by differences in the sample population and/or stage of disease. Furthermore, immature MAIT cells in thymus, cord blood and neonatal blood have higher frequencies of CD161^lo^ MAIT cells and many TRAV1‐2^+^ CD161^+^ cells in thymus and neonatal blood are not MAIT cells.^16^ These data suggest that MAIT cells can modulate CD161 expression, and caution should be applied in interpreting data that relies on the use of TRAV1‐2 and CD161‐specific mAbs to identify MAIT cells in disease settings. While the vast majority, but not all, of TRAV1‐2^+^ CD161^HI^ cells in the DN and CD8α^+^ T cell compartments are MAIT cells, this is a result of their high frequency in healthy individuals. In situations where MAIT cell frequencies are reduced, such as with age,^42^, ^45^ or in disease settings,^36^, ^37^, ^40^, ^44^, ^1^ the proportion of non‐MAIT cells in surrogate marker‐defined populations will increase. Under these circumstances, caution is required in measuring MAIT cell frequency and functional characteristics because the residual cells when MAIT cells are depleted will be enriched for other T cells.

Our data clearly demonstrates that the TRAV1‐2^+^ CD161^+^ surrogate markers are inadequate to identify CD4^+^CD8^−^ MAIT cells, as most of the former cells do not bind to MR1‐5‐OP‐RU tetramer. The identity of the non‐MAIT cells within this population is unclear, but CD161 is also expressed by IL‐17 producing CD4 (Th17) and CD8 (Tc17) T cells,^60^ some of which are likely to randomly express TRAV1‐2. Moreover, some of these cells may belong to the CD4^+^, TRAV1‐2^+^ population of CD1b‐restricted T cells that recognize the Mycobacterium tuberculosis (Mtb)‐derived lipid glucose monomycolate (GMM; GEM T cells) which can also express CD161.^61^ This may especially be a problem in the setting of Mtb infection.

Most MR1 tetramer^+^ MAIT cells are either DN or CD8α^+^ and of the CD8α^+^ subset, many lack CD8β and instead express CD8αα homodimers.^3^, ^17^ Our data suggest that these subsets are phenotypically similar, although minor differences in surface‐molecules such as NKG2D were observed. While CD8α^+^β^+^ MAIT cells develop directly in the thymus, CD8α^+^β^−^ MAIT cells appear to be more mature, appearing in the periphery but not thymus.^16^, ^62^ The physiological role of CD8 co‐receptor usage (whether as CD8αα homodimers or CD8αβ heterodimers) by MAIT cells is unclear, although a recent study suggested that CD8α^+^ MAIT cells had greater cytotoxic function than CD8α^−^ MAIT cells.^47^ The putative CD8 binding site of MR1 is highly conserved with human MHC class I^63^ and several MAIT cell clones have been shown to require CD8 expression for MR1‐mediated activation.^39^ We also demonstrated that 6‐FP‐reactive MAIT cells express high levels of CD8,^14^ suggesting that this coreceptor may modulate responsiveness to low‐affinity Ags.

The CD4^+^CD8^−^ MAIT cells appear to be distinct from other MAIT cells in terms of CXCR6, CD56, NKG2D and NKG2A expression and they produce more IL‐2 than other subsets. In a previous study in mice, CD4^+^ MAIT cells were found to be enriched in lymph nodes and absent in lung, suggesting different tissue homing characteristics for these cells.^19^ We have previously shown immature MAIT cells in thymus express CD4 either in the absence of CD8α, or as CD4^+^CD8α^+^ DP.^16^ This raises the possibility that the CD4^+^CD8α^−/+^ MAIT cells in adult blood are recent thymic emigrants, although the fact that these do not decline with age in blood seems inconsistent with this possibility given the decline in thymic function with age. Furthermore, we previously showed that human thymic CD4^+^ MAIT cell precursors (stages 1 and 2) were incapable of cytokine production,^16^ in contrast to the CD4^+^ fraction from blood which we show here is clearly capable of producing cytokines. Moreover, a recent study also phenotypically compared MAIT cell subsets defined by CD4 and CD8 expression.^64^ That study used the surrogate phenotype of CD161^HI^ TRAV1‐2^+^ to identify MAIT cells, with some but not all of the findings also checked against MAIT cell subsets defined using MR1‐5‐OP‐RU tetramers. While there were several findings that aligned closely between our study and the Kurioka *et al*. study, there were also some important differences. In particular, in the Kurioka *et al*. study,^64^ the CD4^+^ subset was observed to produce both IL‐4 and IL‐13, in contrast to the CD8^+^ and DN subsets which did not produce these cytokines. The CD4^+^ subset was also reported to produce less IFNγ than the CD4^−^ subsets in that study.^64^ In contrast, we failed to detect production of IL‐4 and IL‐13 by CD4^+^ MAIT cells, and we found no significant difference in IFNγ production by CD4^+^ MAIT cells. Perhaps the most likely reason for this discrepancy is that we used MR1‐5‐OP‐RU tetramer to identify the CD4^+^ MAIT cells for cytokine production, whereas the Kurioka et al^64^ study used the surrogate markers CD161^HI^ TRAV1‐2^+^ to define MAIT cells for these experiments. Thus, it may have been non‐MAIT CD4^+^ T cells, which we have shown are abundant in the CD4^+^ CD161^++^ TRAV1‐2^+^ population, that were responsible for these differences in cytokine production in the previous study.^64^ Taken together, CD4^+^ MAIT cells are somewhat distinct from the other subsets; however, they do not appear to be able to produce the Th2 type cytokines IL‐4 and IL‐13. While CD4^+^ MAIT cells might be ignored because they are typically a minor subset of MAIT cells in humans, it is worth reiterating that they are nonetheless present in similar numbers in human blood as Type‐I NKT cells.

The vast majority of MAIT cells had an effector memory cell surface phenotype, and were RORγt^+^ and T‐bet^INT^. This profile is in line with their proinflammatory phenotype and propensity for IFNγ, TNF and IL‐17A production. Of note, in one of twelve donors, there was a subpopulation of MAIT cells with high expression of T‐bet suggesting some level of plasticity in T‐bet expression, aligning with recent reports on MAIT cell functional heterogeneity. For example, it has been shown that liver MAIT cells have a higher propensity for IL‐17A production in response to IL‐7 compared to their peripheral blood counterparts.^5^ Likewise, MAIT cells in the female genital mucosa exhibit a bias toward IL‐17A and IL‐22 production.^21^ The functional implications of this are unclear, although it seems reasonable that MAIT cells located at different anatomical locations might provide a tailored response depending on the local environment. Furthermore studies investigating the extent of transcription factor plasticity and functional consequences will be important. Indeed, in a lung infection mouse model, using *Salmonella* Typhimurium, mouse MAIT cells were shown to skew toward T‐bet expression postinfection.^65^ Whether MAIT cells can be polarized toward T_h_2‐like, TFH‐like or Treg‐like phenotypes (through expression of master transcription factors GATA‐3, Bcl‐6 and FOXp3, respectively) may also have significant biological implications. Examining transcriptional profiles of MAIT cells in disease settings, as well as in tissue resident cells, should provide insights into their functional roles. Moreover, analysis of MAIT cell TCR‐repertoire in these settings will also be important. Indeed, previous reports suggest that the TCR repertoire can influence microbe‐reactivity^52^, ^66^ and differential tissue distribution.^10^ This may be due to differing Ag‐reactivities, and our data provides a potential mechanism for how TRAJ‐gene usage can influence this indirectly by shaping the CDR3β repertoire. Further studies are required to fully understand the contribution of distinct TCR‐α and TCR‐β chains toward MAIT cell functional diversity.

Using MR1‐5‐OP‐RU tetramers, we have confirmed that MAIT cells are highly abundant, albeit variable in frequency, in adult blood donors. Interestingly, we found a positive correlation between the frequency of MAIT cells and both Type I NKT cells and Vδ2^+^ γδ T cells in the peripheral circulation, suggesting they may be regulated by similar environmental and/or genetic factors. In contrast, in mice, MAIT cells appear to compete with NKT cells because CD1d‐deficient NKT cell‐deficient mice exhibit increased numbers of MAIT cells.^16^ Despite these seemingly opposing effects, these findings suggest that MAIT cells and NKT cells share some common developmental or homeostatic factors. We have also demonstrated that MAIT cell frequency is modulated by age, expanding in the first 2–3 decades of life, followed by a gradual decline beyond this stage. There is evidence that expansion of these cells in early life is driven by microbial colonization^9^, ^16^; however, it is unclear why circulating MAIT cell proportions do not peak until the third decade of life. Likewise, the reason for a gradual decline in MAIT cell frequency with aging is unknown. Of note, this has also been reported for Vδ2^+^ γδ T cells^67^ and NKT cells,^68^ again suggesting that these cells may be regulated by common age‐related factors. Interestingly, CD4^+^CD8^−^ MAIT cells showed no correlation between frequency and age, suggesting that they may be ontogenically distinct from other MAIT cells. Nonetheless, these results highlight the importance of appropriate age‐matching of healthy control and disease cohorts when analyzing the frequency of MAIT cells and other innate‐like T‐cell subsets. Whether the decline in MAIT cells renders the elderly more susceptible to infectious disease warrants further investigation.

Taken together, while TRAV1‐2 and CD161 have been useful for identifying and enriching DN and CD8α^+^ MAIT cells from healthy blood samples, their use is not definitive as they may miss some MAIT cells, and may falsely identify other cells as MAIT cells. This may especially be a problem when examining disease cohorts or young or elderly individuals where MAIT cells are numerically low. Thus, MR1‐5‐OP‐RU tetramers detect all MAIT cells, including all subsets defined by CD4 and CD8 coreceptors, and currently provide the most accurate method of identifying MAIT cells in cellular samples.

## Methods

### Human samples

Healthy human blood buffy coats were obtained from the Australian Red Cross after approval from the University of Melbourne Human Research and Ethics Committee and donor informed consent provided (1035100). Young human peripheral blood samples (donors ranged from 5 days to 14 years of age) were obtained from the Royal Children's Hospital (RCH), Victoria, Australia with RCH Human Research Ethics Committee Approval (Ref 24131 G). Peripheral blood mononuclear cells were isolated by standard density gradient (Ficoll‐Paque Plus, GE Healthcare Life Science) and analyzed fresh or cryopreserved for subsequent analysis.

### Cell surface staining

Human PBMC were stained in PBS with LIVE/DEAD Fixable Far Red (Thermofisher Scientific) for 15 min at room temperature. Human Fc‐block (BD Biosciences) was then added at 500 ng/test for a further 15 min at room temperature. Cells were washed once and stained in PBS + 2% FBS for 30 min at room temperature with anti‐surface marker mAb as listed in Supplementary table 1 and human MR1‐5‐OP‐RU or MR1‐Ac‐6‐FP BV421 tetramer (streptavidin‐BV421 from Biolegend). Cells were then washed twice before avidin and biotin blocking (Dako). Cells were then stained for a further 30 min at room temperature with human CD1d‐PBS44 BV605 tetramer (streptavidin‐BV605 from BD Horizon). Cells were washed twice and subsequently either subjected to intracellular transcription factor staining (see below) or fixed with 2% paraformaldehyde for 10 min at room temperature. Cells were analyzed immediately by flow cytometry using an LSRFortessa (BD Biosciences).

### Intracellular cytokine staining

Cells were surface stained as described above with LIVE/DEAD Fixable Far Red (Thermofisher Scientific) and anti‐surface mAb above prior to fixation and permeabilization using 2% paraformaldehyde and 0.3% saponin (BD Biosciences). In brief, cells were fixed using 2% paraformaldehyde for 20 min at room temperature, washed once and then stained in 0.3% saponin overnight at 4 degrees with anti‐cytokine mAb as listed in Supplementary table 1. Cells were then washed twice with PBS and resuspended in PBS + 2% FBS, prior to flow cytometric analysis using an LSRFortessa (BD Biosciences).

### Intracellular transcription factor staining

Cells were stained with a viability dye and surface mAb/tetramers as above. Cells were then permeabilized using a Fix/Perm kit (eBiosciences, FoxP3 kit), according to manufacturer's instructions. In brief, cells were permeabilized for 30 min on ice, washed twice and then stained in permwash for 45 min on ice with anti‐transcription factor mAb as listed in Supplementary table 1. Cells were then washed twice in permwash and resuspended in PBS + 2% FBS. Cells were analyzed immediately by flow cytometry using an LSRFortessa (BD Biosciences).

### Flow cytometry data analysis

All flow cytometric data were analyzed using Flowjo software (Treestar). T cells were gated as CD3^+^ lymphocytes as determined by FSC‐A *versus* SSC‐A after doublet exclusion and removal of dead cells, B‐cells (CD19) and monocytes (CD14; Supplementary figure 8).

### MAIT cell stimulation assays

For intracellular cytokine staining based experiments, Freeze/thawed human PBMCs were cultured in RF10 complete media (RPMI‐1640 (Invitrogen, Life Technologies) supplemented with 10% (v/v) FBS (JRH Biosciences), 2% (v/v) Penicillin (100 U mL^−1^), Streptomycin (100 μg mL^−1^), Glutamax (2 mmol L^−1^), sodium pyruvate (1 mmol L^−1^), nonessential amino acids (0.1 mmol L^−1^), HEPES buffer (15 mmol L^−1^), pH 7.2–7.5 (all from Invitrogen, Life Technologies) and 2‐mercaptoethanol (50 μmol L^−1^, Sigma)), for 7 h in the presence of 10 ng mL^−1^ PMA (Sigma) and 1 μg mL^−1^ Ionomycin (Sigma). Brefeldin A (Sigma) was added for the final 6 h of culture, prior to harvesting cells and performing intracellular cytokine staining as described above.

For cytometric bead array based experiments, MAIT cells were enriched using MR1‐5‐OP‐RU tetramers as previously described.^14^ In brief, fresh, healthy human PBMCs were stained with anti‐TRAV1‐2 FITC (Biolegend) for 30 min at 4°C prior to staining with MR1‐5‐OP‐RU PE tetramers. Cells were then magnetically enriched using MACS anti‐PE microbeads (Miltenyi). After enrichment, cells were surface stained as above with LIVE/DEAD Fixable Near Infrared (Thermo‐Fisher Scientific) anti‐CD4 BV421, ‐CD8α APC (both BD Biosciences), CD8β PE‐Cy7 (eBiosciences) ‐CD14 APC‐Cy7 and ‐CD19 APC‐Cy7 (both BD Pharmingen). MAIT cell subsets were then FACS‐sorted using an ARIA III (BD Biosciences; example purities shown in Supplementary figure 3). Cells were seeded at 2 – 15 × 10^3^ cells/well and then cultured for 24 h in 50 μL RF10 complete media supplemented with 10 ng mL^−1^ PMA (Sigma) and 1 μg mL^−1^ Ionomycin (Sigma). After culture, supernatants were harvested and analyzed for cytokine levels using cytometric bead array.

### Cytometric bead array

Cytometric bead array flex sets were purchased from BD Biosciences and experiments performed as per manufacturer's instructions with an exception that 1/10 the amount of beads and detection reagents were used, and FACS buffer was used in place of official BD wash buffer (as determined by previous in‐house titration experiments). In brief, 10 μL of sample was incubated with 10 μL of bead reagent cocktail for 2 h at RT in the dark. Ten μL of PE‐detection reagent was then added and incubated for a further 2 h at RT. Beads were then washed with 200 μL of FACS buffer, resuspended in 50 μL of FACS buffer and acquired immediately on an LSRII. Data were analyzed using Flowjo Software (Treestar) and Graphpad Prism.

### Human MR1 and CD1d tetramers

Biotinylated human MR1‐5‐OP‐RU tetramers were produced in house (University of Melbourne, VIC, Australia) as previously described.^12^ Human CD1d tetramers were produced in house using a mammalian expression system. The human CD1d extracellular domain was designed with a C‐terminal BirA tag and a 6 × His tag (amino acid sequence at the C terminus: GSGLNDIFEAQKIEWHEHHHHHH). The human CD1d gene as well as the human β2‐microglobulin (β2m) gene were produced (Thermofisher Scientific) and subcloned into separate pHLsec vectors.^69^ Human CD1d‐β2m proteins were expressed by cotransfection of HEK293S GnTI^−^ cells with the plasmids pHLsec‐CD1d‐BirA‐His6 and pHLsec‐β2m.^69^ Purification of CD1d–β2‐microglobulin heterodimers was achieved by immobilized nickel affinity followed by size‐exclusion chromatography. CD1d‐β2m heterodimeric monomers were enzymatically biotinylated with BirA biotin ligase. Biotinylated monomers were loaded with α‐galactosylceramide analogue PBS44^70^ overnight at room temperature at a 3:1 molar ratio of lipid:protein. Tetramers were then prepared using streptavidin‐BV605 (BD Horizon) at a molar ratio of 1:4.

### TCR‐α chain deep sequencing

Healthy human PBMCs were stained with MR1 tetramer/mAb cocktails as above and MAIT cells sorted using an ARIA III flow cytometer (BD Biosciences). MAIT cells were defined as viable CD14^−^, CD19^−^, TCRγδ^−^, CD3ε^+^, TRAV1‐2^+^, MR1‐5‐OP‐RU tetramer^+^ lymphocytes. RNA was immediately isolated using an RNAEasy plus kit (QIAGEN) as per manufacturer's instructions. cDNA was produced using VILO SS RT kit (Thermofisher) as per manufacturer's instructions. After primer optimization, TCR‐α cDNA was amplified by standard polymerase chain reaction (PCR; Eppendorf) using 35 cycles with an annealing temperature of 52°C TRAV1‐2_int and TRAC_int primers from single cell multiplex primer sets.^71^ Amplified DNA was then sequenced using Illumina Miseq with Nextera XT library preparation and 2 × 250 bp cycle (v2) sequencing chemistry as per manufacturer's instructions. At least 260 000 paired‐end reads were obtained for each amplicon. Read pairs were stitched using PEAR, TRAC masking was performed using cross_match and sequence similarity matching to TRAJ genes was performed using BLAST+.

### Transient transfection with TCR constructs

TCR constructs were produced and used to transfect HEK293t cells as previously described.^14^

### Statistical analysis

All graphs were produced using Graphpad Prism. For box and whisker plots, boxes display lower quartile, median and upper quartile. Whiskers display minimum and maximum values, “+” refers to mean. Median value is displayed in text above each plot. Sequence logos were produced using the Seq2logo web server^72^ an unclustered Shannon format in the absence of pseudocounts. The size of each amino acid is proportional to its frequency. Amino acid coloring is based on side chain chemical properties; (red, acidic [DE]; blue, basic [HKR]; black, hydrophobic [ACFILMPVW]; green, neutral [GNQSTY]).

## Conflict of Interest

The authors declare no conflicts of interest.
