# Supplementary material for: Human blood MAIT cell subsets defined using MR1 tetramers
Source: Immunol Cell Biol. 2018 Mar 25;96(5):507–25. doi: 10.1111/imcb.12021 (PMC6446826; doi:10.1111/imcb.12021)

Supplementary Information

Human blood MAIT cell subsets defined using MR1 tetramers

Authors: Nicholas A. Gherardin[1,2], Michael N.T. Souter[1], Hui-Fern Koay[1,2], Kirstie M. Mangas[1], Torsten Seemann[3], Timothy P. Stinear[1], Sidonia B.G. Eckle[1], David P. Fairlie[4,5], David S. Ritchie[6,7], Paul J. Neeson[6], Daniel G. Pellicci[1,2], Adam P. Uldrich[1,2], James McCluskey[1] and Dale I. Godfrey[1,2,*]

Affiliations:

[1]Department of Microbiology and Immunology, Peter Doherty Institute for Infection and Immunity, University of Melbourne, Parkville, Victoria 3010, Australia.

[2]ARC Centre of Excellence in Advanced Molecular Imaging, University of Melbourne, Parkville, Victoria 3010, Australia.

[3]Life Sciences Computation Centre, Victorian Life Sciences Computation Initiative, Carlton, Victoria 3053, Australia.

[4]Division of Chemistry & Structural Biology, Institute for Molecular Bioscience, The University of Queensland, Brisbane, Queensland 4072, Australia.

[5]ARC Centre of Excellence in Advanced Molecular Imaging, University of Queensland, Queensland 4072, Australia.

[6]Cancer Immunology Program, Peter MacCallum Cancer Centre, East Melbourne, Victoria 3002, Australia.

[7]Department of Medicine, University of Melbourne, Parkville, Victoria 3010, Australia.

*Corresponding author

Supplementary Table 1: List of anti-human monoclonal antibodies

**Epitope**	**Clone**	**Fluorophore(s)**	**Manufacturer**
CD3ε	UCHT1	AF700, BUV395	BD Pharmingen
CD4	SK3	AF700, BUV496	BD Pharmingen
CD4	SK3	BV510	Biolegend
CD8α	SK1	BUV805	BD Pharmingen
CD8 β	2ST8.5H7	APC, PE-Cy7	BD Pharmingen
CD14	MoP9	APC-Cy7	BD Pharmingen
CD19	2J25C1	APC-Cy7	BD Pharmingen
CD26	BA-5b	PE-Cy7	Biolegend
CD27	O323	BV785	Biolegend
CD28	CD28.2	PE-Cy5	Biolegend
CD45RA	HI100	PerCP-Cy5.5	Biolegend
CD56	5.1H11	BV605	Biolegend
CD62L	DREG-56	APC	BD Pharmingen
CD127	eBioRDR5	PE-Cy7	eBiosciences
CD158a (KIR2DL1)	HP-MA4	APC	Biolegend
CD158b (KIR2DL2-3)	DX27	PE	Biolegend
CD158f (KIR2DL5/S5)	UP-R1	APC	BD Pharmingen
CD161	HP-3G10	PE-Cy7, BV650	Biolegend
CD218 (IL-18Rα)	H44	PE	Biolegend
NKG2A	Z199	PE-Cy7	Beckman Coulter
NKG2D	1D11	PE-Cy7	Biolegend
NKp30	P30-15	PE	BD Pharmingen
NKp44	P44-8	AF647	Biolegend
CCR5	2D7/CCR5	FITC	BD Pharmingen
CCR6	11A9	APC	BD Pharmingen
CCR7	3D12	PE-Cy7	BD Pharmingen
CXCR6	13B 1E5	BV421	BD Pharmingen
TRAV1-2	3C10	APC	Biolegend
TCRγδ	11F2	FITC, PE-Cy7	BD Pharmingen
TCRVδ1	TS8.2	FITC	ThermoFisher
TCRVδ2	B6	BV711	Biolegend
IFNγ	XMG1.2	BV650	BD Pharmingen
TNF	MAb11	BV421	BD Pharmingen
PLZF	Mags.21F7	PE	eBiosciences
T-bet	4B10	PE	eBiosciences
RORγt	AFKJS-9	PE	eBiosciences
GATA-3	TWAJ	AF488	eBiosciences

Supplementary Figure Legends

Supplementary Figure 1. Validation of anti-CD8α and anti-CD8β co-staining on MAIT cells. (a) Flow cytometric pseudo-colour plots from 1 representative healthy donor PBMC sample showing CD8α expression (top panel) or CD8β expression (lower panel) on MAIT cells stained with either anti-CD8α (left), anti-CD8β (middle) or both (right). **(b)** Box and whisker plots showing the proportion of MAIT cells positive for CD8α or CD8β when stained with either one or two anti-CD8 anti-bodies (n=4). **(c)** Box and whisker plots showing the MFI of (i) CD8α or (ii) CD8β on MAIT cells or conventional T cells when stained with either one or two anti-CD8 anti-bodies (n=4).

Supplementary Figure 2. Control Tetramer staining on MAIT cell subsets. Flow cytometric plots from one representative donor from 4 showing MR1 tetramer staining with tetramer controls on αβ T cell co-receptor-based subsets.

Supplementary Figure 3. Phenotyping of healthy donor G73. Flow cytometric pseudo-colour plots showing the percentage of MAIT cells in total αβ T cells from healthy donor G73 as well as the co-receptor distribution and CD161 expression on TRAV1-2^{+} MR1-5-OP-RU^{+} αβ T cell.

Supplementary Figure 4. CD161^{INT} cells confound surrogate phenotyping of CD4^{+} MAIT cells. (a) FACS plots showing example gating strategy for single cell sorting of CD4^{+} MAIT cells. **(b)** Table showing CDR3α amino acid sequences of TCRα chains derived from CD161^{HI} or CD161^{INT} TRAV1-2^{+} CD4^{+} T cells as illustrated in A.

Supplementary Figure 5. MAIT cell co-receptor subset analysis. (a) Flow cytometric pseudo-colour plots showing CD161 and CD26 expression on total αβ T cells (n=1) MAIT cells (n=4) from 4 donors, representative of 2 experiments. **(b)** Flow cytometric pseudo-colour plots showing the percentage of MAIT cells in total αβ T cells from healthy donor G64. **(b)** Flow cytometric pseudo-colour plots showing the expression of CD161, CD26, IL-18Rα, CD27, CD28 and T-bet on MAIT cells in healthy donor G64.

Supplementary Figure 6. Representative FACS-Sort purities for MAIT cell subsets. Representative FACS plots from one donor showing reanalysis of FACS-sort-purified MAIT cell subsets that were used in PMA/Ionomycin stimulation assays.

Supplementary Figure 7. TCR-β chain mutagenesis of alternate TRAJ-gene^{+} TCRs. (a) Histogram overlays showing MR1-5-OP-RU tetramer staining of 293T cells transiently transfected to express 3 TCRs and their β-chain mutant counterparts. (**b**) Bar graph showing % decrease in tetramer staining for each cell line upon β-chain mutation. Results from E are representative of 2 independent experiments carried out in duplicate conditions.

Supplementary Figure 8. Flow Cytometry Gating Strategy of human MAIT cells from PBMC. Flow cytometric pseudo-colour plots showing gating strategy of MAIT cells from human PBMCs. Lymphocytes are first gated using FSC-A and SSC-A parameters. Doublets are then removed with FSC-A and FSC-h parameters. Dead cells, B cells and monocytes are removed by excluding viability^{+} cells, CD19^{+} and CD14^{+}

cells respectively, all in the one dump channel. αβ T cells are gating using CD3 and TCRγδ mAb. MAIT cells are then defined at TRAV1-2^{+} MR1-5-OP-RU tetramer^{+} cells.

Supplementary Figure 1

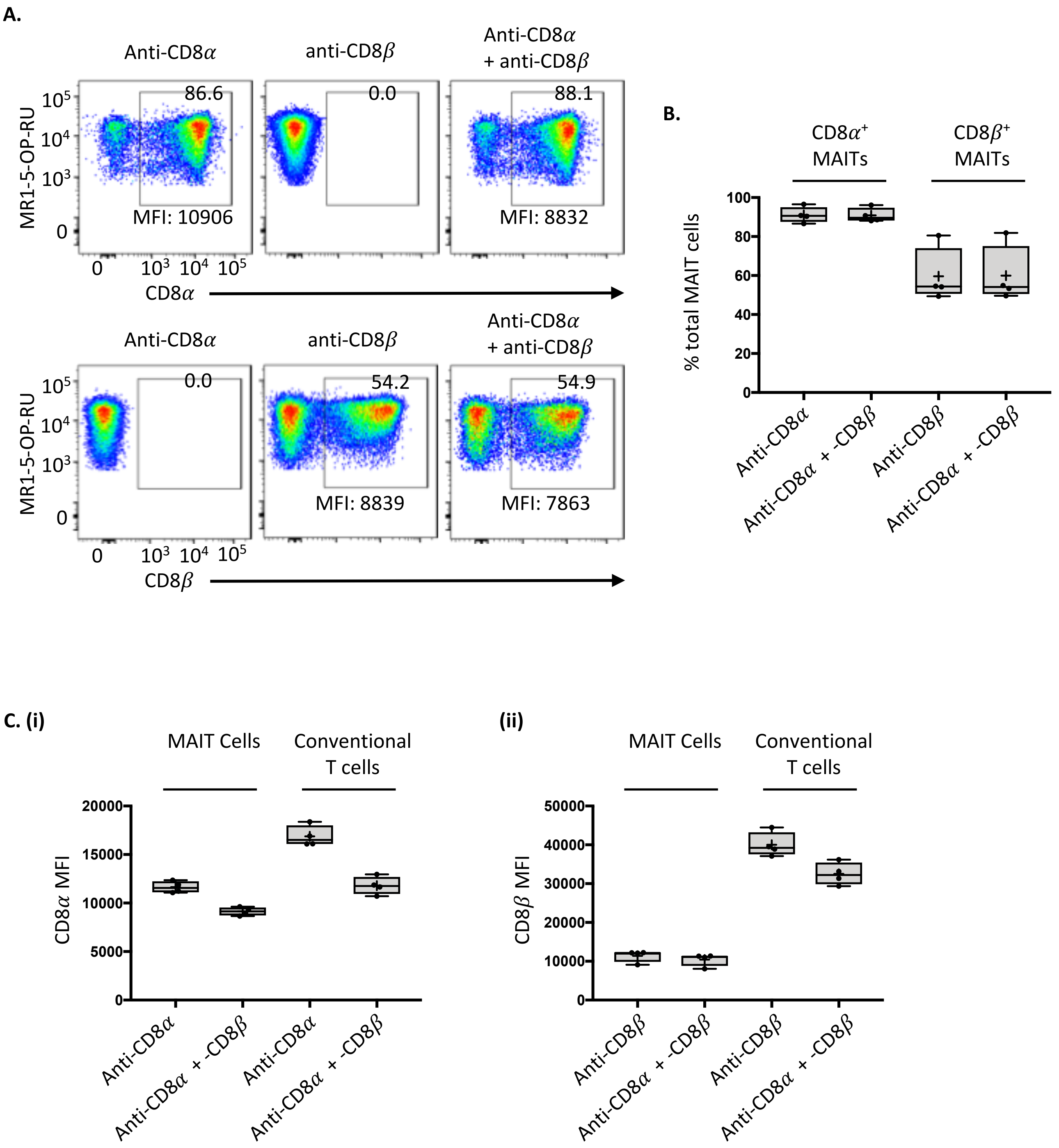

Supplementary Figure 2

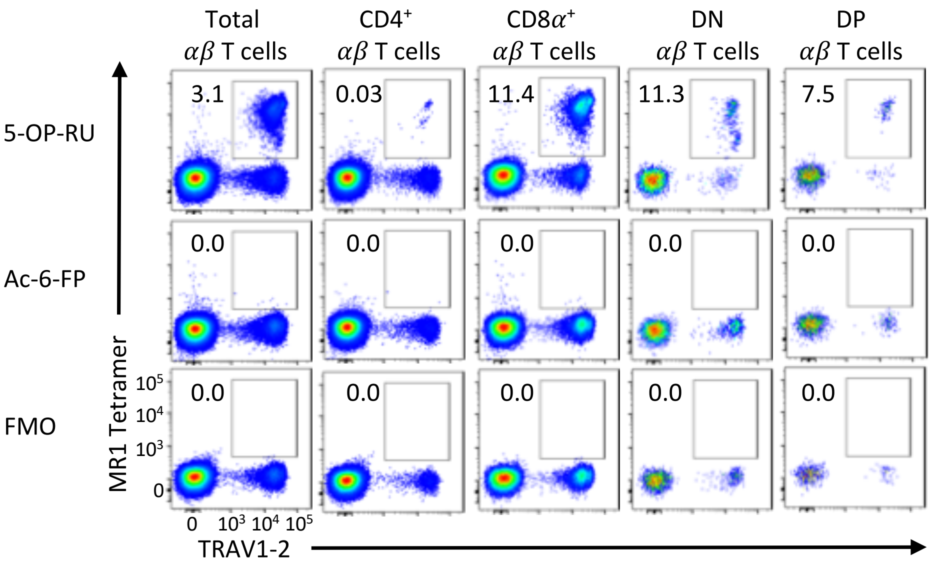

Supplementary Figure 3

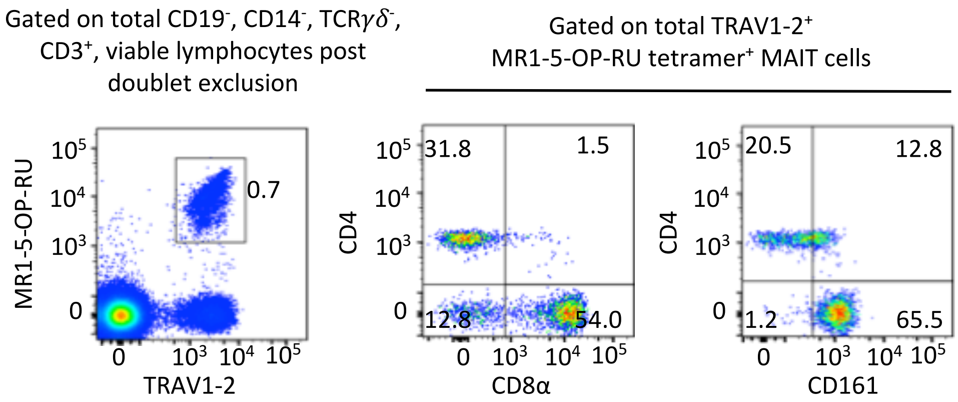

Supplementary Figure 4

A.

Gated on total CD19⁻, CD14⁻, TCR$\gamma\delta^-$, CD3⁺, CD4⁺, viable lymphocytes post doublet exclusion

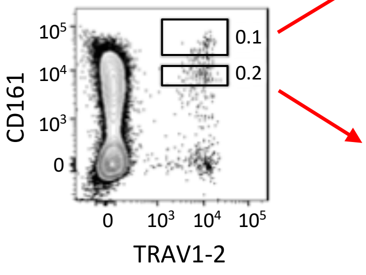

B.

TRAV	CDR3α	TRAJ	Freq.
1-2	CAVMDSNYQLIW	33	1/8
1-2	CAVMDSNYQLIW	33	1/8
1-2	CAVVDSNYQLIW	33	1/8
1-2	CAAMDSNYQLIW	33	1/8
1-2	CAVMDSSYKLIF	12	3/8
1-2	CAVLDSSYKLIF	12	1/8

TRAV	CDR3α	TRAJ	Freq.
1-2	CAVMDSNYQLIW	33	2/7
1-2	CACMDSNYQLIW	33	1/7
1-2	CAVPTNDYKLSF	20	1/7
1-2	CAVRLTGGFKTIF	9	1/7
1-2	CAVRDAGDDKIIF	30	1/7
1-2	CAVKQMEYGNKLVF	47	1/7

Supplementary Figure 5

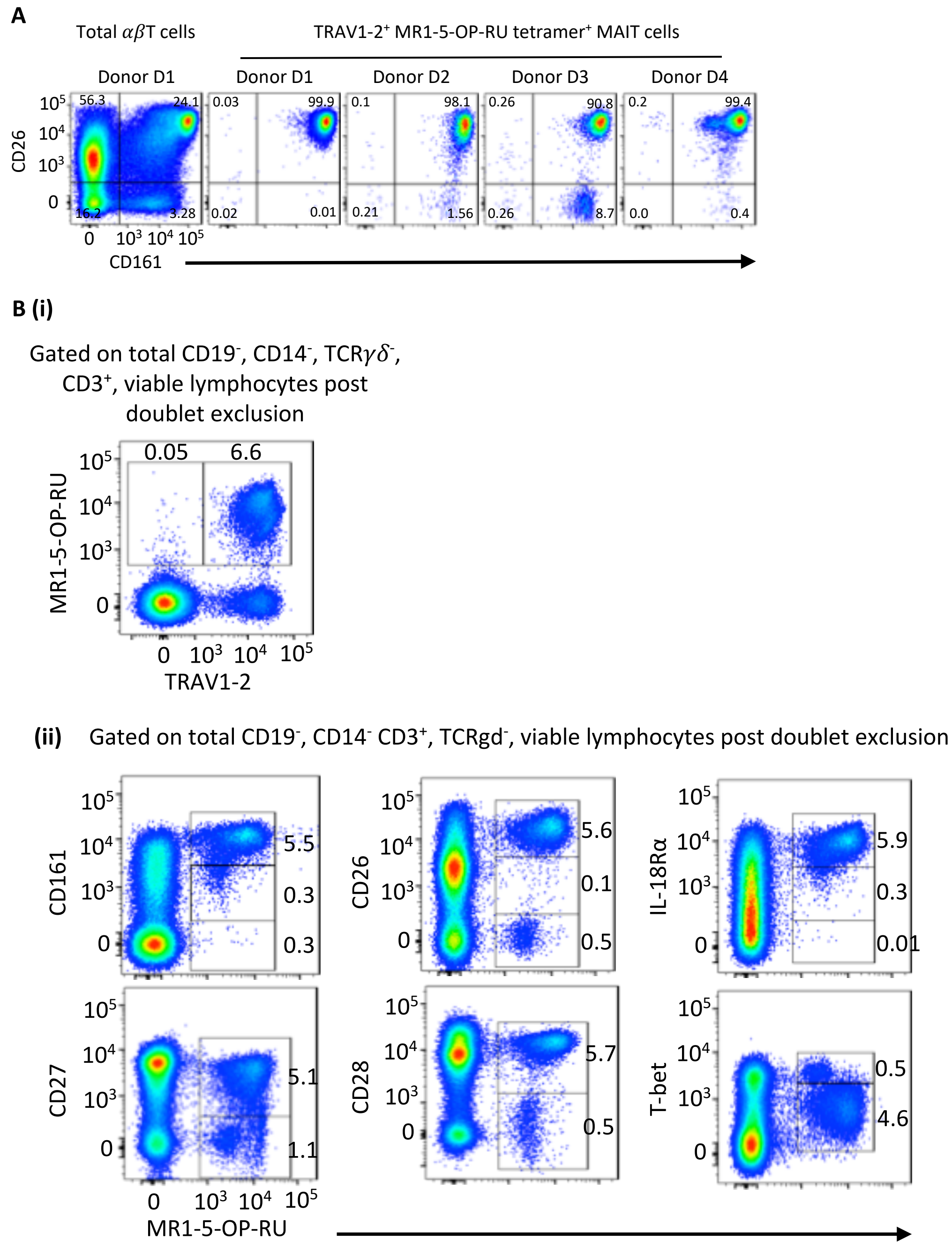

Supplementary Figure 6

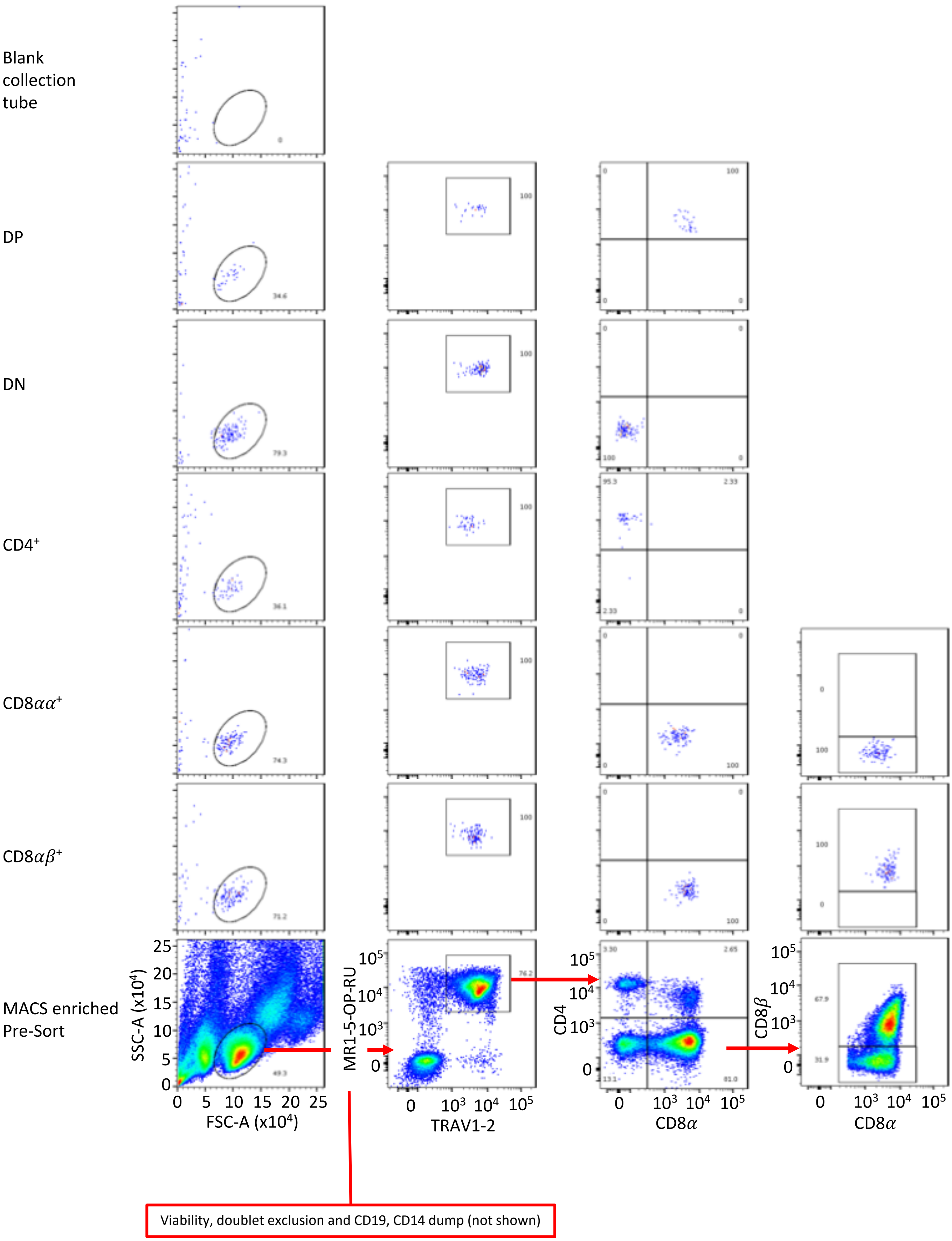

Supplementary Figure 7

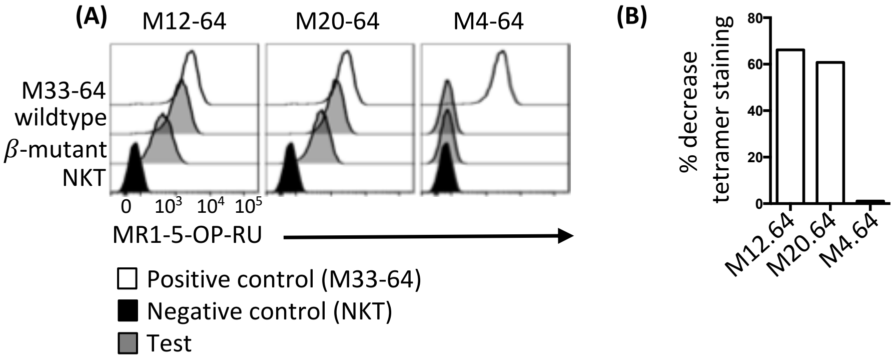

Supplementary Figure 8

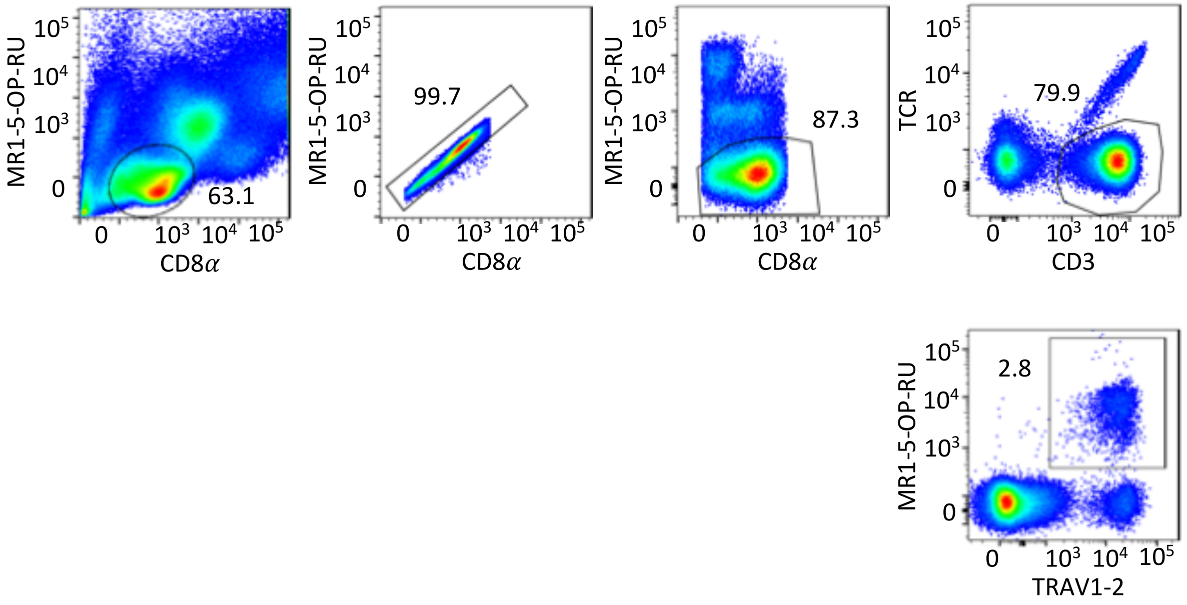

Supplement: Supplementary file 1 [file IMCB-96-507-s001.pdf]
